# Young children copy cumulative technological design in the absence of action information

**DOI:** 10.1038/s41598-017-01715-2

**Published:** 2017-05-11

**Authors:** E. Reindl, I. A. Apperly, S. R. Beck, C. Tennie

**Affiliations:** 1 University of Birmingham, School of Psychology, Edgbaston, Birmingham B15 2TT UK; 2 University of Tübingen, Department for Early Prehistory and Quaternary Ecology, Burgsteige 11, 72070 Tübingen, Germany

## Abstract

The ratchet effect – the accumulation of beneficial changes in cultural products beyond a level that individuals could reach on their own – is a topic of increasing interest. It is currently debated which social learning mechanisms allow for the generation and transmission of cumulative culture. This study focused on transmission, investigating whether 4- to 6-year-old children were able to copy cumulative technological design and whether they could do so without action information (emulation). We adapted the spaghetti tower task, previously used to test for accumulation of culture in human adults. A baseline condition established that the demonstrated tower design was beyond the innovation skills of individual children this age and so represented a culture-dependent product for them. There were 2 demonstration conditions: a full demonstration (actions plus (end-)results) and an endstate- demonstration (end-results only). Children in both demonstration conditions built taller towers than those in the baseline. Crucially, in both demonstration conditions some children also copied the demonstrated tower. We provide the first evidence that young children learn from, and that some of them even copy, cumulative technological design, and that – in line with some adult studies – action information is not always necessary to transmit culture-dependent traits.

## Introduction

Humans’ capacity to spread across the planet and to reach out beyond its boundaries has often been explained with their ability to produce cumulative culture, i.e., to accumulate changes in cultural traits beyond a level that individuals can reach on their own^1, 2^. These changes entail both improvements and deterioration, as well as changes that have no effect on trait efficiency. However, it is our capacity to accumulate *beneficial* modifications over time – a phenomenon labelled the *ratchet effect* ^3, 4^ – that is thought to be among the key characteristics setting us apart from other animals, including culture-bearing species such as chimpanzees and orangutans^3^. Identifying the cognitive processes underpinning the ratchet effect will help us understand more about how human culture has evolved.

Researchers have begun to experimentally investigate the cognitive mechanisms involved in cumulative culture: Methods have been developed to simulate cumulative cultural evolution in the laboratory, e.g., behavioural experiments using the transmission chain paradigm^5–12^ or virtual tasks investigating the effects of social learning in groups^13, 14^. These studies show that human adults readily exhibit a ratchet effect. In conjunction with other experimental studies^15–17^ and modelling approaches^18, 19^ these findings suggest that one crucial prerequisite for the ratchet effect is the capacity for high-fidelity social transmission, alongside a capacity for innovations^20^ and other relevant cognitive mechanisms^21^.

High-fidelity social learning is essential for the transmission of cumulative culture in both the social and the technological domain^22^. The current study focuses on the technological domain as material culture has played a crucial role for the evolution of our species^8, 23, 24^. Over evolutionary time, our ancestors increasingly relied on technology to solve problems, which was likely both the result of and the driving force for the evolution of ever more faithful cultural transmission mechanisms. This co-evolution of faithful social transmission and material culture has resulted in the creation of the human “technological niche”^25^.

Which social learning mechanisms enable the occurrence of a ratchet effect? Many social learning mechanisms such as stimulus or local enhancement are capable of supporting culture over extended time, yet they have been argued to be of insufficient fidelity and bandwidth to *accumulate* culture^26, 27^. In these cases, the learning mechanisms draw the learner’s attention towards a stimulus/location, after which the behaviour in question is acquired through individual learning. The acquisition of the trait is thus more an individual response than a faithful copy. Consequently, the range of traits that a learner can acquire by this combination of low-fidelity social learning and individual learning is limited to those that the learner can actually invent individually^4^. Cultural traits that are too complex or unlikely to be reinvented individually – traits that we label *culture-dependent traits* – cannot – by definition – be acquired by mechanisms that only harness the power of individual learning (such as stimulus and local enhancement)^26^. Instead, culture-dependent traits need high-fidelity copying to be transmitted, e.g., copying of the actions involved and/or end-results produced. It has been argued, and demonstrated, that cumulative culture can be transmitted via imitation, i.e., transmission where action copying plays a role^1, 3, 4, 9, 12–14, 17, 28^. Whether *emulation*, i.e., learning only about effects (or “results”) in the environment^29^, can ever be sufficiently faithful to sustain a ratchet effect is still debated^6, 7, 10, 17, 27, 30^.

The general capacity for high-fidelity social learning is within the human cognitive repertoire from an early age^31^: Before the end of their first year, infants are able to copy novel actions^32^; by 1 year, they flexibly switch between emulation and imitation^33, 34^ and by the end of their second year, children imitate even causally irrelevant actions^35–40^ (*overimitation* ^41^) – a propensity argued to be highly adaptive in an environment of cognitively opaque cultural artefacts and skills^24^ (in fact, the tendency to overimitate extends well into and throughout adulthood)^42–45^. Given the special role high-fidelity copying plays for cumulative culture, young children clearly already possess some crucial cognitive prerequisites for acquiring and transmitting cumulative culture.

However, so far no study has investigated children’s ability to learn from or even copy cumulative culture in the technological domain. This is surprising as the acquisition of technological skills is an important form of cultural transmission in our species, responsible for the vast amount and complexity of technology accumulated today. Therefore, our study examined children’s capacity for copying *cumulative technological design*. We define technological design as a material cultural product created by a sequence of instrumental actions, i.e., actions which “bring about a tangible, functional outcome”^22^, whereby the actions are causally – i.e., non-arbitrarily – linked to this outcome. The creation of technological design is a subtype of *instrumental skills* ^22^: Instrumental skills are those that achieve functional outcomes by either arbitrary (e.g., typing in a number combination to unlock a phone) or non-arbitrary (e.g., levering to open a box) actions (with arbitrary actions requiring the learner to pay relatively more attention to the actions compared to the results of the demonstration). Technological design represents the group of products that are created by non-arbitrary actions only, and thus are inherently more results-focused.

Previous studies on children’s social learning often aimed to differentiate between imitative and emulative learning^31, 46^, thus testing children’s motivation and ability to act on and/or manipulate parts of the environment based on demonstrations. None of these studies tested for the recreation of technological design. Nevertheless, they provide important insights into children’s copying behaviour, many of which are relevant to understanding the acquisition of (cumulative) technological design. The studies that come closest to testing children’s capacity to copy culture-dependent traits investigated children’s ability to copy *social conventional* ^22^, as opposed to instrumental, acts. Social conventional acts are usually those in which the relationship between the outcome and (parts of) the actions is not causal, but arbitrary (thus conventional)^34, 37, 41, 47, 48^ (e.g., driving on the left or the right side of the road, tapping the side of a box before opening it). Acts can also be interpreted as social conventional if they contain obviously inefficient actions^33, 49^ (e.g., switching on a light with your forehead rather than your hand) or if the start states and end states of these acts do not differ^50^. These studies show that from their second year of life children are able to copy novel conventional acts. As some of these behaviours were not shown spontaneously by the children (i.e., without having received a demonstration), e.g., turning on a light with the head^49^, these behaviours might even represent social-conventional culture-dependent traits for children.

While previous studies have tested for children’s ability to copy *social conventional* culture-dependent traits, the question of children’s capacity for learning from and copying *technological* culture-dependent traits (cumulative technological design) remains unanswered. Conclusions drawn from studies involving social conventional actions may not apply to the technological domain, not least because in the latter both imitation and emulation may be important^51, 52^. For example, technological demonstrations allow the learner to gain information by attending to endstates as well as intermediate states. Therefore, in contrast to social conventional demonstrations, learners could focus only on the outcome and then reproduce it using their own means (so-called endstate emulation^29^). Technological tasks also allow learners to combine action and results copying, resulting in greater transmission fidelity than that which copying a single type of information alone would be able to achieve (“redundant copying”^51^). Thus, social conventional and technological demonstrations differ systematically with regard to the social learning mechanisms that can be involved, which likely has implications for the transmission of cumulative culture.

Young children use imitation and emulation to perform instrumental tasks even before their first birthday^32, 33, 53, 54^. However, the target actions/results in previous studies have only consisted of simple actions/results or combinations of those that participants in baseline conditions were able to invent on their own. Thus, they did not meet the requirements for culture-dependent traits (that no individual can and does show the trait in question without demonstrations). Our study is the first to look at children’s ability to copy culture-dependent traits in the technological domain.

We investigated young children’s ability to copy a technological culture-dependent product (cumulative technological design) and whether children required action information to do so (or whether seeing only the results was sufficient). For this, we adapted the spaghetti tower task previously used to study aspects of cumulative cultural evolution in adults^5^. In contrast to previous studies on children’s social learning in instrumental tasks^15, 46, 53–55^ we showed that the demonstrated trait (building a “tripod” design, see below) was not invented by children spontaneously.

In Pilot study 1 (see Supplementary method: Pilot study 1) we tested children between 4 and 6 years and showed that this age range was suitable for the adapted tower construction task. In order to present children with a culture-dependent product, i.e., a product that “no individual could invent”^26^, we needed to establish what children of that age can achieve on their own given the materials provided: In Pilot study 2 (see Supplementary method: Pilot study 2 and Supplementary Table S1) we examined children’s baseline performance by asking 17 4- to 6-year-olds to build something “as tall as possible” using plasticine and sticks. Based on these data, we created our cumulative technological design: a construction that was not spontaneously invented by the baseline children.

**Figure 1:**
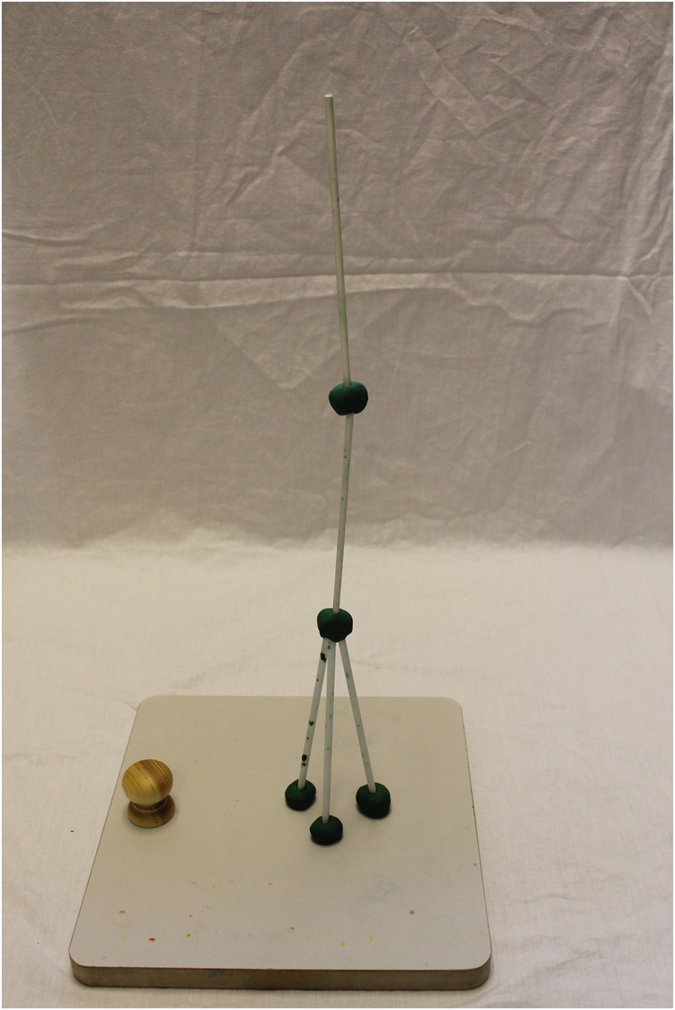
Demonstration tower (tripod). Example of the cumulative technological design children in the demonstration conditions were presented with (h = 46 cm).

In cumulative cultural evolution, changes to a technological product can occur in two domains^21, 56^: In its *complexity*/*design* (in our case the shape of the construction) and in its *efficiency* (the height of the construction). We focused on the design aspect and chose as our cumulative technological design a *tripod* – a tower with a base of three legs arranged in a triangle (rather than, e.g., in a line) and combined at the top with a piece of plasticine (Fig. 1). Cumulative culture is inherently open-ended^56^ and the tripod operationalizes this aspect by being a hierarchical and open-ended cultural product: The order of the building actions is determined through a hierarchy (e.g., “first construct the tower base, then build upwards. For the base, first form the plasticine, then insert the sticks, etc.”); at the same time the number of possible steps is not limited by the task (open-ended). We chose a hierarchical task as “most cultural products are compound products (p. 285)”^57^, requiring a “lengthy sequence of actions […] with each action functionally dependent on previous actions (p. 3801)”^58^. In sum, instead of presenting children with simple actions/results or combinations of those as has been the case in previous studies, we presented children with a *cultural recipe* ^58^.

The tripod represents a superior design compared to other tower designs, as it allows for greater heights to be achieved. Evidence comes from studies using the spaghetti tower task with adults which have identified the tripod as one of the most efficient designs invented by participants^7, 59^. Moreover, Caldwell and colleagues^7^ found a positive relationship between the number of tripod design features that participants’ constructions exhibited and the height of these constructions; such a relationship was absent for a cubic design, suggesting that the tripod allows for greater heights to be achieved, and so comes closer to the notion of open-endedness. These studies also found that participants who were shown a tripod design were themselves able to build constructions that were as tall as^59^ or even significantly taller^7^ than the demonstrated tripod, whereas participants presented with a different (cubic) design were not able to go beyond the demonstrated height. Note that the fact that the tripod is superior to many other designs does not imply that participants choosing the tripod design will always make towers that are taller than other shapes.

Pilot study 2, in which we established children’s baseline performance, also included a second condition (full demonstration condition, hereafter *full demo condition*) in which a new group of children observed the experimenter making a tripod before they built their own construction. Results showed that children between 4 and 6 years were able to copy the tripod from this action plus endstate demonstration, and is thus the first evidence for young children’s ability to copy cumulative technological design (see Supplementary method: Pilot study 2).

The study presented here had three goals: First, to replicate the findings from Pilot study 2: i) that children in a baseline condition would not produce a tripod on their own, and ii) that children in a full demo condition would be able to copy the tripod design. This replication was important because in Pilot study 2 we did not collect the data for the two conditions simultaneously (because by design, baseline data had to be collected first) and thus comparability of these two conditions might have been impaired. In addition, and more generally, the reproduction of novel findings is crucial to enhance their credibility – especially when a study is the first of its kind^60^. Second, we aimed to investigate whether children would also be able to copy cumulative technological design when they lacked information about the actions involved in producing the tripod. For this, we added to the baseline and full demo condition a third condition, an endstate-only demonstration condition (hereafter *endstate-only demo condition*). Lastly, we also introduced small improvements to our general methodology (see Supplementary Method: Pilot study 2). This study contributes to our understanding of human culture in shedding first light onto the ontogenetic origins of how children use social and technical information when acquiring technological culture-dependent traits.

## Results

### Tower height

Across conditions, children’s average tower height was 25.05 cm (*SD* = 11.68 cm), ranging from 0.3 to 45.5 cm. In the baseline, tower height was on average 17.84 cm (*SD* = 10.60 cm, range 0.3–41.0 cm); in the full demo condition, it was 28.07 cm *(SD* = 10.96 cm, range 15–45.0 cm), and in the endstate-only demo condition it was 28.62 cm *(SD* = 10.68 cm, range 2–45.5 cm). Pictures of children’s towers can be found in Supplementary Tables S3–S5, histograms of the heights reached in the three conditions are displayed in Fig. S1.

Overall, sex, age in months, and condition (baseline, full demo, endstate-only demo) together had a clear effect on the height of children’s towers (comparison full-null model: *F*(4, 68) = 5.339, *p* < 0.001). Specifically, tower height was significantly affected by condition (*F*(2, 68) = 7.992, *p* < 0.001; R^2^ = 0.19). Children in the full demo condition built towers that were on average 10.65 cm (*SE* = 3.12; 95% CI [4.43; 16.87]) taller (*p* = 0.001), and children in the endstate-only demo condition built towers that were 10.51 cm (*SE* = 2.98; 95% CI [4.56; 16.45], *p* < 0.001) taller than the towers built in the baseline. Tower height did not differ between the two demo conditions (estimate + SE: −0.14 + 3.02, *t*_68_ = −0.047, *p* = 0.962). Even though at the group level, children in the demo conditions built taller towers than children in the baseline, only 10 out of 52 children in the demo conditions built towers that went *beyond* the maximum height achieved in the baseline. Age also had a significant positive effect on tower height: With each standard deviation increase in age (i.e., 4.31 months), average tower height increased by 2.91 cm (*SE* = 1.25, *t*_68_ = 2.33, *p* = 0.022; R^2^ = 0.07). Sex had no effect on tower height (0.30 + 2.50, *t*_68_ = 0.12, *p* = 0.906). In sum, results showed that older children built on average taller towers than younger children and that children in both demo conditions built taller towers than those in the baseline.

### Tower shape

No child in the baseline made a tripod, thus replicating the finding from Pilot study 2. The most common tower shape was a level-1-tower (see Table 1 for descriptions) in the baseline and a level-2-tower in both demo conditions. Crucial to our research question, children in both demo conditions made tripods: Three children in the full demo and one child in the endstate-only demo condition copied the tripod (three of these children were younger than 5 years). A further five children built smaller versions of tripods (level-2-tripods). Finally, one child in the endstate-only demo condition built a level-4-tripod. Although this tower exceeded the demonstration tripod with regard to height in stick levels (see Table 1), the height in cm did not exceed the tripod height as the child’s tower was slightly crooked. A more detailed analysis of children’s tower heights and shapes can be found in the Supplementary results, as well as Figures S2, S3, and Table S6.

**Table 1:** Distribution of tower height in stick levels and tower shape in the three conditions.

Tower height in stick levels	Tower shape	Shape description	Condition		
			Baseline	Full demo	Endstate demo
Level 4	Level-4-tripod	As Tripod, but additional stick on top			1
Level 4 Total		0/23 (0%)	0/23 (0%)	1/27 (3.7%)	
Level 3	Tripod	Three legs, combined with plasticine, two sticks on top of each other added above		3	1
	Level-3-tower	Three sticks combined vertically on top of each other (at least one stick per level)	2	3	6
Level 3 Total		2/23 (8.7%)	6/23 (26.1%)	7/27 (25.9%)	
Level 2	(modified) Level-2-tripod	small tripod (at least three legs – plasticine – stick)		2	2
	Level-2-tower	Two sticks combined vertically on top of each other (at least one stick per level)	4	6	8
	Other level-2-constructions		1	2	3
Level 2 Total		5/23 (21.7%)	10/23 (43.5%)	13/27 (48.1%)	
Level 1	Level-1-tower	Ball of plasticine with vertical stick on top or two level-1-towers combined with sticks combined at top	6	5	4
	Hedgehog	Ball of plasticine from which several sticks protrude upward and/or sideward	5	2	
	Other level-1-construction				1
Level 1 Total		11/23 (47.8%)	7/23 (30.4%)	5/27 (18.5%)	
Level 0	Horizontal construction	Construction with sticks and plasticine, intentionally built in horizontal fashion	3		
	Plasticine tower	Plasticine-only tower	2		1
Level 0 Total		5/23 (21.7%)	0/23 (0%)	1/27 (3.7%)	

### Similarity to tripod

Participants’ constructions were evaluated by two coders (blind to the hypotheses) who judged the similarity of all constructions to the demonstration tripod using a scale from 1 (*not similar at all*) to 7 (*very similar*). Mean similarity of baseline towers to the tripod was 2.12 (*SD* = 1.08); for full demo towers it was 3.05 (*SD* = 1.44); for endstate-only demo towers it was 3.05 (*SD* = 1.61). Condition had a significant effect on the similarity of children’s towers to the demonstrated tripod, χ^2^ = 7.934, df = 2, *p* = 0.019. Post-hoc Tukey tests showed that towers in the full demo condition were rated significantly more similar to the tripod compared to the similarity of baseline towers to the tripod (*p* = 0.036) and the same pattern was found for the towers in the endstate-only demo condition (*p* = 0.023). Ratings for the towers in the two demo conditions did not differ significantly (*p* > 0.999).

## Discussion

This study investigated 1) whether young children would be able to use social and/or technical information provided by a demonstration of cumulative technological design in a tower construction task in order to improve their tower building skills and 2) whether some of the children would even spontaneously copy the cumulative technological design. Four- to six-year-olds were assigned to one of three conditions (baseline, full demo, endstate-only demo) and were asked to build something as tall as possible out of plasticine and sticks. Children in the full demo condition observed the experimenter build cumulative technological design – a tripod – and children in the endstate-only demo condition were presented with a ready-made tripod. Pilot study 2 had established that the tripod design was not within children’s spontaneous capacities (as indicated by their baseline performance), establishing that the tripod represented cumulative technological design for children that age. We replicated the pilot study by showing that children in the baseline condition did not invent the tripod shape; in addition, they neither reached the demonstrated tripod height nor went beyond it.

We also showed that children in both demo conditions built on average taller towers than children in the baseline, suggesting that children were able to pick up task-relevant information from the demonstration of the culture-dependent product and to improve their performance. This is in line with a recent study on adult social learning showing that access to social information can boost a learner’s performance^13^. Yet, even though towers in the demo conditions were on average taller than baseline towers, there was some overlap in tower height between the conditions. In contrast, with regard to tower *design*, there was a clear difference between the baseline and the demo conditions: there were no tripods built by any child in the baseline. Thus, the difference between baseline and demo conditions with regard to tower height was less dramatic than the difference with regard to tower design.

We found that some children (including 4-year-olds) in both demo conditions copied the specific and efficient shape of the tripod (three legs), even though they were not instructed to do so. Ratings by independent coders confirmed that the towers in the demo conditions were more similar to the demonstrated tripod than were the towers in the baseline. Thus, our study demonstrates for the first time that 4- to 6-year-old children are not only able to use social and technical information to improve their performance, but that some of these children also spontaneously copy an efficient cumulative technological design. Note that we do not claim the ability to copy tripods to be present in all children of this age (though it may be – given that we did not tell children to copy the tripod, but just asked them to make something as tall as possible). As children did not receive an explicit instruction or incentive to copy the tripod, our study was a conservative test of whether children can copy cumulative technological design and so it was even more impressive to find that some children indeed copied the tripod.

Intriguingly, children in the endstate-only demo condition did not perform differently from children in the full demo condition. Despite lacking information about the building process of the tripod, some children in the endstate-only demo condition still copied the tripod, suggesting that they were able to recreate the tripod via *end-state emulation*: That is, children were able to identify the necessary building steps from looking at the tripod and to recreate it through reverse engineering. This is evidence that 4- to 6-year-olds do not necessarily require action information to be able to copy cumulative technological design (i.e., technological culture that is beyond their own spontaneous abilities). Rather, children were able to use emulation as a social learning mechanism to copy the cumulative technological design in our task; imitation of the actions involved proved not necessary.

Even though children were not instructed to copy the tripod, it is worth considering that despite receiving action or end-state demonstrations, only a few children actually reproduced the tripod. A number of factors might explain this result. First, some children might have lacked the motivation to copy. After all, there was no direct incentive for copying the tripod (other than it being tall, of course). In addition, the task was not set in a competitive context, so children might have preferred to not copy, but to realize their own ideas and/or explore the materials. For example, we had a couple of children who – upon receiving the demonstration – acknowledged that the tripod was “a good tower”, but then went on making something completely different; in particular, one boy said at this point: “Oh, this is good, but I will make something else”. Still developing fine motor abilities at this age probably also contributed to the fact that only few children copied the tripod. Finally, difficulties in causal reasoning and insight into the mechanics of the construction task might also have played a role for children’s low copying rates. In general, children did not yet have a full understanding of the basic physics involved in the task, as several children used only little plasticine for the stand and/or too much for the joints further up. With regard to the ability to copy the tripod, it has been shown that 5-year-olds have difficulty using diagonals when building towers and that this is due to a still developing ability to combine two axes of a coordinate system^61, 62^. Given this difficulty, some of our participants might have failed to explicitly recognize the tripod legs as an especially stable tower base. Despite these limiting factors for children’s performance, we still found that children were able to pick up useful knowledge and strategies from the demonstrations, resulting in them making taller towers than the baseline children – and some did indeed successfully copy the tripod design.

One might object that the absence of the tripod in the baseline condition does not necessarily mean that the tripod represents a culture-dependent trait for 4- to 6-year-old children. It might be possible that even though the tripod did not occur in the baseline, it is still within children’s spontaneous abilities, but that environmental and/or motivational reasons prevented children from showing their “true capacities” (building a tripod). We think this is unlikely because 1) children were sufficiently motivated to do the task (they were aware that they could win a sticker; the vast majority of children used the full building time; we encouraged children throughout the task to make their construction “even taller”), 2) they had sufficient material at their disposal, and 3) the finding that children in the baseline condition did not spontaneously build a tripod was confirmed by two independent studies (Pilot study 2 in the Supplementary material and the study presented here). In addition, children’s limited knowledge about the physics involved in the task (see above) makes it further unlikely that children could invent the tripod on their own.

However, one might also argue that the fact that children in the baseline condition did not build a tripod is due to the specific instructions we gave: We merely asked children to build “something tall”, and so might have increased children’s awareness of the height aspect only without also directly addressing the importance of the shape aspect. So given different instructions (e.g., to build “something tall and sturdy”), one might expect some baseline children to actually make tripods. However, we would not expect this to happen as in our view the instruction to build something tall does not deemphasize the shape aspect. This is because height and shape are closely connected: When aiming to build a tall construction, one almost automatically needs to take the shape aspect into consideration, as some shapes will be more suitable for building a tall tower than others. Nevertheless, we acknowledge that we currently do not know how baseline children would perform if given different instructions. Future studies are needed to address this question. However, the current study was not designed to answer questions related to the instructions, but to test children’s capacity of making a tripod given an instruction to make something tall and given the presence or absence of a tripod demonstration.

Experimental studies on the production or transmission of cumulative culture need to determine carefully what they define as culture-dependent traits. Previous studies^5, 15^ have not included control conditions, making it “difficult to conclude with certainty that these experiments have demonstrated true cumulative culture”^63^. While not giving us absolute certainty, including a baseline condition helps determine whether a trait (e.g., building a tripod) can be easily generated by or tends to be beyond the spontaneous capacity of individuals (see e.g. refs 10 and 12). Our study is the first to include a baseline condition in the tower construction task. This allowed us to 1) assess children’s spontaneous, asocial learning capacities, 2) identify a tower representing a culture-dependent trait for 4- to 6-year-olds (tripod), and 3) make meaningful comparisons of the performance in the demo conditions against a baseline performance. Even though we could not fully rule out that some children had previous experience with a similar game, the weak baseline performance (low height, no tripods) is reassuring that the task was sufficiently novel (and difficult) for our participants. We thus established that the tower task is suitable for studying the transmission of culture-dependent traits in young children: 4- to 6-year-olds are old enough to possess the necessary fine motor abilities to carry out the task, but are still young enough to be able to benefit from social information.

Our results are in line with some adult studies showing that emulation is capable of transmitting information about culture-dependent traits^6, 7, 10^. However, it has also been argued^3, 26^ (and demonstrated experimentally in adult studies^9, 13, 14, 17^) that seeing only an end product may *not* be sufficient for transmitting cumulative culture. One factor possibly influencing whether culture-dependent traits can be transmitted via emulation is the cognitive transparency of the product in question^6, 13^; however, the mechanisms of this dependency are still unclear. It seems that cultural traits exhibiting sufficient cognitive transparency can be acquired via emulation, because seeing the end product allows learners to reproduce them via reverse engineering (e.g., building paper planes^6^, spaghetti towers^7^, baskets^10^ or plasticine-and-stick tripods (this study)). Similarly, Want and Harris^31^ proposed that if children have sufficient knowledge about the affordances and properties in a task, its “solution can be emulated”^p12^, otherwise children would need to revert to copying actions. The transmission of cultural products that are cognitively more opaque (e.g., novel weight-carrying devices^9^, virtual fishing nets^14^, foam handaxes^17^, stone tools^8^ or complex (virtual) totems^13^) seems to require additional information about the movements of the objects involved and/or of the bodily actions of the demonstrator, so that learners would be able to copy the trait via object-movement reenactment (a fine-grained version of emulation learning ref. 64), action copying (roughly: imitation ref. 65), or both^51^. Therefore, it might be possible that culture-dependent traits can be differentiated by their cognitive transparency, with this transparency in turn influencing whether emulation is sufficient to transmit the traits.

Our findings raise the question whether young children, at least some of whom our study has shown to be capable of copying cumulative technological design, would also be able to transmit and maintain cumulative culture among themselves (i.e., across “generations”). Future studies applying the transmission chain paradigm^66^ could explore whether cumulative technological design can be maintained along a chain of children or whether it would disappear (compare with the method applied in Morgan *et al*.^8^). Another question is whether children would also be able to *produce* culture-dependent traits themselves, i.e., whether they could successively build upon ever better solutions, thus exhibiting a ratchet effect. It might be that children find this innovative aspect of producing culture-dependent traits difficult. Support for this thought comes from our finding that participants in our demo conditions were not able to go beyond the height of the demonstrated tripod and that only one participant (in the endstate-only demo condition) built a level-4-tripod. Furthermore, research on innovation in children demonstrated surprisingly low innovation rates with regard to making tools^67^ or inventing strategies to retrieve more rewards from a puzzle-box^68^: Children well into their primary school years seem to struggle with inventing (better) solutions to new tasks. This might imply that groups of young children might not yet be able to show a ratchet effect as continuing limitations on their ability to innovate represents a critical bottleneck for the development of a capacity for producing culture-dependent traits [see also refs 69 and 70]. Again, the transmission chain method will prove useful for investigating whether generations of children can also produce cumulative technological design by adding innovations to existing solutions and then transmitting these. These studies will shed more light on the developmental origins of humans’ unique cultural abilities.

## Method

### Participants

Seventy-three children (34 boys) between 4 years 2 months and 5 years 8 months (*M*_age_ = 5 years 0 months*, SD* = 4.31 months) were tested in nursery schools and a science museum in a metropolitan area in the UK. The ethnic composition was 59% Caucasian, 27% Asian, and 14% Black. Written informed consent was obtained by participants’ parents or guardians prior to the study. Children were randomly assigned to the baseline (*n* = 23, 43.5% male), full demo condition (*n* = 23, 56.5% male), or endstate-only demo condition (*n* = 27, 40.7% male); comparable numbers of children from each testing site were represented in each condition. There were no differences in the distribution of age (Kruskal-Wallis-test, χ^2^(2) = 0.963, *p* = 0.618) between conditions. Another two children were tested but excluded from the analysis as they did not answer the control question correctly. Ethical approval was granted by the University of Birmingham, UK, STEM Ethical Review Committee. The study was carried out in accordance with the approved guidelines.

### Procedure

Children were tested individually by the same female experimenter (E). Both were sitting on the floor, at a low table (h = 15 cm). To confirm children’s understanding of the concept “taller”, testing started with a control question for which children were shown two Playmobil® giraffes of differing sizes and asked to indicate which animal was taller. Next, children were told that they would play a game in which they could win a sticker. E said: “The game is to build something that is very tall, as tall as you can make it”. E showed children 30 white plastic sticks (“lollipop sticks”; length = 15 cm, diameter = 4.5 mm) and 70 g of green plasticine and said: “You can use these things to help you build it. You can do anything you like with these things to try to make something very tall. You can use all of these [*E pointed to lollipop sticks*] and all of this [*pointed to plasticine*]. Also, with this [*E took plasticine*] you can do things like this [*E tore one third off of the ball*] or this [*E tore another third off, then rolled it*].”

In the demo conditions, E then said: “Before you start, let me show you what I did earlier!” In the full demo E built the tripod (~50–60 sec). Upon completion, she said “Finished!” and looked at the tripod for 5 sec. She then placed the tripod on a box ~20 cm behind the table, where it was available for inspection throughout the trial. In the endstate-only demo, E fetched a board with a ready-made tripod from behind a barrier next to her and placed it on the table. She looked at it for 5 sec and moved it to the box. The rest of the instructions in the demo conditions was the same as the instruction children in the baseline were given: E said: “You don’t have much time to build something that is as tall as possible”; this was to induce them to be quick as their building time was only 6 min. E then encouraged children to start building.

During the building phase, E took measurements of children’s towers using a folding rule attached to the table, each time participants made an addition to their construction which increased its height and if the construction was standing on its own (i.e., children did not stabilize it with their hands). The measurement was done by visual judgement, a procedure shown to be sufficiently reliable (Supplementary method: Pilot study 3, Supplementary Table S2). When the time was up, children were not allowed to touch the construction anymore. Children who held their construction in their hands were asked to place it on the table and those who stabilized it with their hands were told to let go. Towers that could not stand on their own had to be placed horizontally on the table. Tower height was measured again with a loose folding rule held right next to the construction. Once each participant had left the room, E took pictures of the construction (one from each side, one from above).

### Coding and statistical analysis

We were interested in whether children in the demo conditions would be able to copy the demonstration tripod. In addition, we investigated whether children in the demo conditions would build taller towers than children in the baseline condition, and how similar children’s constructions were to the demonstrated tripod. For this, we measured three variables: *tower height* (height of the tallest construction a participant built), *tower shape* of the tower with the maximum height, and *similarity to tripod* (similarity of a child’s construction to the tripod).

#### Tower height

For each participant, tower height was measured several times: throughout and at the end of the trial. This allowed us to identify each participant’s tallest construction, even if the construction did not “survive” until the trial end, e.g., because children disassembled it or because it collapsed due to being too instable or because children tried to further modify their construction. Since instances of tower collapses often resulted from the fact that we encouraged children to use the full building time (i.e., even when children announced they were finished we encouraged them to continue building in order to ensure equal construction time among participants), we measured tower height continuously to ensure that we made a fair evaluation of children’s performance. Consequently, for some children tower height represented the height of the tower which stood on the table at the end of the trial, whereas for other children tower height represented the height of a tower that they had built during the trial, but that did not survive until the trial end.

We analyzed whether tower height differed between conditions, using a multiple regression including condition (baseline, full demo, endstate-only demo) as the predictor, sex (dummy-coded), and age in months (covariate) as control variables, but no interaction as we did not predict one. Prior to fitting the model, we confirmed that tower height and age had symmetrical distributions. We z-transformed age to a mean of zero and a standard deviation of 1 in order to facilitate the interpretation of the coefficients. We checked the following model diagnostics: normal distribution and residuals plotted against fitted values (to check for homoscedasticity of residuals), DFFits and DFBetas, Leverage, Cook’s distance, Generalized Variance Inflation Factor, and Levène’s test of equal error variances. There were no obvious deviations from the model assumptions. To determine the effect of condition, we compared the fit of the full model with the fit of a model lacking condition as a predictor. The model and the diagnostics were run in R (version 3.2.3 ref. 71), the Generalized Variance Inflation Factor was calculated with the function “vif” of the R package “car”^72^. Sample size for this analysis was 73; the alpha level for all analyses in this study was 0.05.

#### Tower shape

The shape of the tallest tower was coded offline by E.R. based on photos and video stills. First, we first determined the height structure of the tower in what we labelled *stick levels* (Table 1, first column): We counted how many sticks were vertically combined on top of each other (“combining” meaning two sticks joined vertically with a piece of plasticine, while an overlap of up to half the length of a stick was allowed). This allowed us to group the towers into four categories: *level-0-constructions* (towers that were smaller than the height of one stick, e.g., towers lying on their side or constructions consisting only of plasticine); *level-1-constructions* (constructions with one (or more) sticks placed vertically into a plasticine base); *level-2-constructions* (comprising any constructions in which two sticks were combined on top of each other); and – applying the same logic – *level-3-* and *level-4-constructions* (the tripod thus fell into the level-3-constructions group). We further grouped the towers within each stick level category based on their shape, resulting in one to three shape categories per stick level (Table 1, columns 2 and 3).

#### Similarity to Tripod

Similarity to tripod was coded based on the method used by Caldwell & Millen^5^. Two raters, blind to the research hypotheses, coded pictures of all the towers (i.e., towers at trial end and – for children whose tallest tower did not survive until trial end – additionally the tallest tower from throughout the trial) with regard to their similarity to the demonstrated tripod, using a scale from 1 (*not similar at all*) to 7 (*very similar)*. Scaled points 2–6 were not labelled specifically. For each participant, raters were given one picture of the participant’s construction, which was to be compared with a picture of the demonstration tripod (presented in five pictures, to show also the slight, but unavoidable variance of the demonstrated tripods) and asked “How similar is this [image] to the constructions on the 5 pictures?” The ratings of the two coders correlated significantly (*r* = 0.828, *p* < 0.001) and the strength of the relationship between the two ratings was similar to the one in Caldwell & Millen^5^.

To determine whether the towers in the demo conditions were rated as more similar to the tripod than the towers in the baseline, we fitted a Linear Mixed Model^73^ into which we included condition as a fixed effect and random intercepts for participant and rater (to account for the fact that each rater and each participant contributed more than one data point). We also included a random slope for condition (manually dummy-coded) on rater^74^. The model was fitted using the function lmer of the R-package lme4^75^. To allow for a likelihood test, we fitted the model using Maximum Likelihood (rather than Restricted Maximum Likelihood ref. 76). We checked for normal distribution and homoscedasticity of the residuals by visually inspecting a qq-plot and the residuals plotted against the fitted values and found no obvious violation of these assumptions. The significance of condition was determined by comparing the full model against a reduced model (lacking the variable condition) by a likelihood ratio test (R function anova with argument test set to “Chisq”^77^). Post-hoc Tukey tests were carried out using the R package multcomp^78^. The sample size for this model was a total of 178 ratings made on 89 towers (two ratings per tower).

### Data accessibility

The datasets generated and analysed during the current study are available at the UK Data Service ReShare repository, https://dx.doi.org/10.5255/UKDA-SN-852706.

## Electronic supplementary material

Supplementary material
