# Supplementary material for: Young children copy cumulative technological design in the absence of action information
Source: Sci Rep. 2017 May 11;7:1788. doi: 10.1038/s41598-017-01715-2 (PMC5431834; doi:10.1038/s41598-017-01715-2)
Supplement: Supplementary file 1 — Supplementary material [file 41598_2017_1715_MOESM1_ESM.pdf]

Supplementary material

Young children copy cumulative technological design in the absence of action information

Reindl[1]*, E., Apperly[1], I. A., Beck[1], S. R., & Tennie[1,2], C.

[1]University of Birmingham, School of Psychology, Edgbaston, Birmingham B15 2TT, UK

[2]University of Tübingen, Department for Early Prehistory and Quaternary Ecology, Burgsteige 11, 72070 Tübingen, Germany

Contents:

- Supplementary Method: Pilot study 1

- Supplementary Method: Pilot study 2
    - Supplementary Table S1

- Supplementary Method: Pilot study 3
    - Supplementary Table S2

- Supplementary Method: Main study
    - Table S3 (Pictures of towers in baseline condition, main study)
    - Supplementary Table S4 (Pictures of towers in full demo condition, main study)
    - Supplementary Table S5 (Pictures of towers in endstate-only demo condition, main study)
    - Supplementary Fig. S1 (Maximum tower heights in the three conditions of the main study)
- Supplementary Results: Main study
    - Supplementary Fig. S2
    - Supplementary Table S6
    - Supplementary Fig. S3

Supplementary Method: Pilot study 1

Identifying an appropriate age range for the construction task

We conducted extensive pilot work in order to determine the age window for which the tower task was most appropriate. Initial pilot work (not reported here), in which we tested slightly different construction tasks (e.g., building a horizontal construction or a tower from materials such as Play Doh®, wooden sticks or tape) indicated that children below 4 years of age found any of these tasks very difficult. This was likely because of children's difficulties in understanding the goal of the game, their still developing understanding of the physics involved in the task (e.g., aspects related to gravity and mass), children's still developing fine motor abilities, or limited interest in and/or motivation to play the game. Therefore, we concluded that children below 4 years of age would likely be too young for our study.

In Pilot study 1 we tested for the first time our final tower construction task, with 15 children between 3 years 6 months and 4 years 4 months. Again, we found that several children in this age range tended to be too young for the task, as they struggled with fully understanding the physical characteristics of the task, were limited by their still developing fine motor abilities, or sometimes did not seem to understand the task or lacked enough interest to build something as tall as possible. Therefore, we decided the lowest age for which the task was appropriate to be 4 years.

We also collected data from three children aged 5 years 10 months. One child was assigned to the baseline condition and built a level-3-tower. Two children were tested in the demonstration conditions and showed good performance. We concluded from Pilot study 1 that the tower task was most suitable for studies with children between 4 and 6 years of age.

Supplementary method: Pilot study 2

In Pilot study 2, we aimed 1) to explore children's baseline performance in our tower construction task, 2) based on the findings from the baseline condition, to create a demonstration tower (the tripod) which was beyond the spontaneous capacities of children that age, and 3) to investigate whether children in a full demo condition (receiving information about actions and the end results) would be able to copy the cumulative technological design.

The procedure was similar to the procedure of the main study presented in the main manuscript. We asked baseline children to build something that was as tall as possible. This condition informed us about which tower heights and shapes children of this age could achieve without help, and therefore data for this group were collected first. Children in the full demo condition received the same instruction, but before they started, they observed the experimenter (E) build a tower whose complexity they were not able to invent on their own – i.e., that went beyond the achievement shown by children in the baseline. Full demo children were thus presented with cumulative culture (for their age). In order for this tower to represent cumulative culture for the children, we chose a design/shape that never occurred in the baseline: A *tripod* – a tower with three legs supporting two sticks on top of each other (what we refer to as a *level-3-construction*).

Method

Participants

We tested 34 children (15 boys) between 4 and 6 years (M_{age} = 4 years 10 months (4;10), *SD* = 5.14 months, range: 4;1 to 5;9) from a metropolitan area in the UK. The ethnic composition was 53% Caucasian, 29% Black, and 18% Asian. Baseline children (tested first)

were recruited and tested in nursery schools. Children in the full demo condition (tested second) were recruited via advertisements on a local parenting website and on the website of the science museum where the testing for this condition took place. Parents willing for their child to participate gave written informed consent. Seventeen children (six boys) were assigned to the baseline, the other half to the full demo. There were no differences in age ($t(32) = 0.662$, $p = .513$, Cohen's $d = 0.228$) or sex ($\chi^2(1) = 0.477$, $p = .490$) between conditions. Participants were rewarded with a sticker regardless of their success.

Materials

Warm-up game

We used a day-night Stroop task consisting of 24 cards showing a picture of either daytime or nighttime. Children should say "night" when shown the day card, and "day" when shown the night card. We chose this game as pilot work showed that children were motivated to play this game, it required little space and material, and could be adapted to children's skill level. The task was used to familiarize children with E and responses were not recorded.

Tower task

The materials were the same as in the main study.

Procedure

The procedure and instructions were the same as in the main study, with the exception of five aspects, which we changed for the main study: First of all, Pilot study 2 did not yet include the control question for the understanding of "taller than".

Second, in the instructions for the tower task, we used the expressions "high" and "higher than" as opposed to "tall" and "taller than" (e.g., "Try to make something as high as possible."). After the completion of the study we considered that "high" and "higher than"

could be somewhat ambiguous, and therefore we changed these expressions for the main study.

Third, the instruction for the full demo condition included a turn-taking aspect. That is, participants were told that the game involved turn-taking, so that E would start first and that the child’s task was to watch her. When the time was over, it would be the child’s turn to “build something that it as high as possible”. We decided to remove the turn-taking aspect from the instructions for the main study to make the instructions more comparable to the one used in the baseline.

Fourth, in the full demo condition, we did not yet use a second table behind the construction table where we transferred the demonstrated tripod to. Instead, we placed the tripod in the floor. As we noticed that some children compared the height of their towers with the height of the tripod without taking into consideration that their bases are not equally tall, we decided to use a second table with equal height for the main study.

Finally, we measured tower height only at the end of the testing session as we had not yet developed a means to measure tower height during the trial. That is, for each child there was only one measurement for tower height in cm, representing the height of the tower that was on the table at the end of the session (final tower height). Note that this was not necessarily the tallest tower children built throughout the session.

Coding and analysis

As in the main study, we took four measurements (online and offline). All analyses were carried out with IMB SPSS Statistics 22.

Tower height

Tower height represented the height of participants' construction at the end of the trial. Therefore, tower heights did not necessarily reflect the maximum height achieved by the child, as in some cases towers crashed and could only partly be rebuilt in the remaining time. This was especially the case for the full demo: For 9 out of 17 children, the towers with the maximum height crashed or were disassembled and rebuilt. Thus, at the end of the trial, towers with a smaller height than the maximum height achieved were measured, which potentially underestimated children's performance. In the baseline, there were only four children for whom the final tower did not also represent the tower with the maximum height. Although we were not able to establish the exact height of towers that did not "survive" until the end of the trial, we were able to determine their height in stick levels (see results) from stills of the videos.

Tower shape

As in the main study, we classified the shape of the towers in an offline analysis. For children for whom the final towers were also the tallest towers, we based this classification on the photos taken at the end of the trial. For children for which the tallest tower was built throughout the session but did not "survive" until its end (because it crashed or because children disassembled it; baseline: n = 4, full demo: n = 9) we coded both the final tower (using the photos taken at the end of the trial) and the tower with the maximum height (using stills from the video). The classification procedure was the same as in the main study.

Similarity to tripod

We established the similarity of all of the children's towers to the demonstrated tripod, using the same procedure as in the main study. The same raters as in the main study rated pictures of children's towers with regard to their similarity to the tripod, using a scale from 1

(*not similar at all*) to 7 (*very similar*). For children whose final tower was the tower with the maximum height, final towers were rated; for children whose final tower was not the one with the maximum height, both the final tower and the tallest tower from throughout the trial were measured (as long as video was available).

The ratings of the two coders correlated significantly (r = .565, p < .001); and the strength of the relationship between the two ratings was similar to the one in Caldwell & Millen[5]. To determine whether the towers in the full demo condition were rated more similar to the demonstrated tripod than the towers in the baseline, we fitted a Linear Mixed Model using the same procedure as in the main study. The sample size for this model was a total of 94 ratings made on 47 towers (two ratings per tower).

Results

Tower height

Across conditions, mean final tower height was 19.85 cm (*SD* = 11.68 cm), ranging from 3.5 to 44 cm. In the baseline, mean tower height was 15.56 cm (*SD* = 8.18 cm), ranging between 3.5 and 33.5 cm. In the full demo, mean tower height was 24.15 cm (*SD* = 13.24 cm), ranging from 4 to 44 cm.

To investigate whether tower height differed between the baseline and full demo condition, we conducted an analysis of covariance (ANCOVA) with final tower height as the dependent variable (DV), condition (baseline, full demo) as the independent variable (IV), and age (in months) as a covariate. All assumptions for ANCOVA were met. We found significant main effects for condition, $F(1,31) = 8.473$, $p = .007$, partial $\eta^2 = .215$, and age, $F(1,31) = 9.710$, $p = .004$, partial $\eta^2 = .239$ on tower height. Older children built significantly taller towers than younger children. On top of the age effect, children in the full demo

condition built significantly taller towers than those in the baseline, supporting the idea that children were able to benefit from a demonstration of cumulative culture.

The difference in tower height between conditions might have been even more pronounced had we measured children's towers not only at the end of the trial, but also throughout (as we did in the main study). Although we did not collect continuous data on the height (in cm) of towers throughout the trial, we recorded height in stick levels for every construction children made during the trial. Therefore, in a separate analysis, we first investigated tower height in stick levels for the *final* towers. We found that in the baseline, the tallest towers made were level-2-constructions. In the full demo condition, the tallest towers were level-3-constructions. Since the variable *stick levels* was not normally distributed, we used a Mann-Whitney U test to determine whether there was a statistical difference between conditions with regard to final tower height in levels. Results showed that there was a statistically non-significant trend of final towers in the full demo condition reaching higher levels that those in baseline (U = 89.00, p = .057, Cohen's d = 0.795). Next, we analyzed the stick level of towers with the *maximum* height and found a significant difference between conditions (U = 53.50, p = .001, Cohen's d = 1.357).

Tower shape

No child in the baseline made a tripod (Supplementary Table S1). The most common tower shapes in the baseline were hedgehogs and other level-2-constructions (both shapes were made by 4 out of 17 children). In the full demo condition, the most common tower shape was a level-2-tower (6/17 children). Out of the six children in the full demo condition who built a level-3-construction, two children (aged 4;7 and 4;11 years) produced a very similar tripod to the one they saw demonstrated. A further two children in the full demo condition built modified tripods with more than three legs. Three additional children in the full demo condition built smaller tripods with a level height of two sticks. Across conditions, the four

level-3-tripods represented the tallest constructions in the sample (40.5, 42, 43, and 44 cm; *M* = 42.37 cm, SD = 1.49 cm: compared to the rest of the full demo condition participants with *M* = 18.54 cm, SD = 9.40 cm, and compared to the baseline participants with *M* = 15.56 cm, SD = 8.18 cm).

Table S1. *Height and shape of children's towers with the maximum height.*

Tower height in levels	Tower shape	Shape description	Condition<br>Baseline	 Full demo
Level 3	Tripod	Three legs, combined with plasticine, two sticks on top of each other added above		2
	Modified tripod	Tripod with more than three legs		2
	Level-3-tower	Three sticks combined vertically on top of each other	1	1
	Other level-3-construction			1
Level 3 total			1/17 (5.9%)	6/17 (35.3%)
Level 2	Level-2-tripod	small tripod (three legs – plasticine – stick)		3
	Level-2-tower	Two sticks combined vertically on top of each other (at least one stick per level)	1	6
	Other level-2-construction		4	
Level 2 total			5/17 (29.4%)	9/17 (52.9%)
Level 1	Level-1-tower	Ball of plasticine with vertical stick on top or two level-1-towers combined with sticks combined at top	1	2
	Hedgehog	Ball of plasticine from which several sticks protrude upward and/or sideward	4	
	Other level-1-construction		2	
Level 1 total			7/17 (41.2%)	2/17 (11.8%)
Level 0	Crashed construction	Any vertical construction (e.g. level-2-tower) which failed to stand alone	2	
	Horizontal construction	Construction with sticks and plasticine, intentionally built in horizontal fashion	1	
	Plasticine tower	Plasticine-only tower	1	
Level 0 total			4/17 (23.5%)	0/17 (0%)

Similarity to tripod

Condition had only a marginally significant effect on the rated similarity of children's towers to the demonstrated tripod, $\chi 2 = 3.049$, df=1, p=.081. Please note that this analysis also included the final towers for children whose tower with the maximum height was built earlier in the trial and did not survive until its end. This was the case for 13 children, 9 of which were in the full demo condition. When we excluded these (smaller) towers and reran the analysis, we did find a significant effect of condition on the rated similarity of children's towers to the demonstrated tripod, $\chi 2 = 4.545$, df=1, p=.033.

Discussion

This pilot study established that the tower construction task is a suitable means for studying cumulative culture in children aged 4 to 6 years. Upon observing a demonstrator building a cumulative technological design, i.e., an artefact that children this age would not be able to make on their own, children produced towers that were on average taller than those built by a baseline group who did not have the opportunity for cultural learning. In addition, some of the children in the full demo condition were able to produce a faithful copy of the tripod. These findings thus provide the first evidence that 4-year-old children are able to copy cumulative technological design.

Since this study was our first attempt to investigate acquisition of cumulative culture in children, and since it is – to the best of our knowledge – the first study in general investigating at what age children become able to acquire cumulative technological design, we aimed to replicate our findings in the main study. Another reason for carrying out a replication was the fact that the data for the two conditions in Pilot study 2 were not collected simultaneously; furthermore, data for the two conditions were collected at different places (baseline in nurseries, full demo condition at Science Museum). This might have made the two groups of children somewhat less comparable. A third reason to carry out our main study

was that we aimed to include several improvements to the study design and improvements: We chose a more interactive warm-up game (spinning tops), we removed the turn-taking aspect in the full demo, we changed the wording in the instructions to “tall” and “taller than”, we included a control question to ensure children’s understanding of “taller than”, we used a second table placed behind the construction table, and finally and most crucially, we introduced a simple method that allowed us to measure tower height throughout the trial without interrupting children’s building process. Another major reason for carrying out the main study was that we aimed to include an endstate demo condition to investigate what kind of information children require to benefit from the cumulative culture demonstration.

The raw data and pictures of children’s towers for Pilot study 2 are available upon request.

Supplementary method: Pilot study 3

Establishing the validity of measuring tower height through visual judgement

One of the methodological changes we introduced in the main study was to measure the height of children’s towers continuously throughout the building process, as opposed to only measuring it once at the end of the trial as was done in Pilot study 1. We aimed to measure tower height at any time that children added to their construction a new item which increased the tower’s height and to take the measurement without disrupting the building process.

We placed a stationary folding rule at the side of the table, opposite of where E sat. Whenever a child added to the construction an item that increased tower height, E compared the tower to the folding rule in the back to estimate its height. We conducted six trials in a pilot study to determine how precisely E was able to measure tower height via visual judgement. For this, E first built a tower of a random height and shape, after which she measured its height first by visual judgement and then using a folding rule held directly next to the constructions (as was the method for measuring the towers at the end of the trial). The results showed that measurement by visual judgement was a reliable method, with only minimal measurement error (Table S2). The general procedure for the main study was to take measurements as conservatively as possible. Thus, whenever E was in doubt which of two measurements to take (usually 1 cm apart, e.g., 31.5 or 32.5 cm), she took the higher measurement for the baseline and the lower measurement for the demo conditions, so that she would not artificially increase children’s performance difference between the baseline and the two experimental conditions (because E was not blind to the hypotheses).

Table S2. *Pilot data on the accuracy of measuring tower height by visual judgement*.

	**Estimated height** (measured by visual judgement)	**Actual height** (measured with folding rule)	**Estimation error**
Tower 1	19.5 cm	19.5 cm	0.0 cm
Tower 2	16.0 cm	15.5 cm	0.5 cm
Tower 3	41.0 cm	40.0 cm	1.0 cm
Tower 4	31.0 cm	31.0 cm	0.0 cm
Tower 5	57.0 cm	57.5 cm	0.5 cm
Tower 6	7.0 cm	6.5 cm	0.5 cm

Supplementary method: Main study

Table S3. *Towers of children in the baseline condition, sorted by height of final tower.*

**Subject**	**Age** (in months)	**Sex**	**Final tower**<br>*Picture*<br>*Shape*<br>*Height*	**Tower with maximum height** (if different from final tower)<br>*Picture*<br>*Shape*<br>*Height*
93	59	F	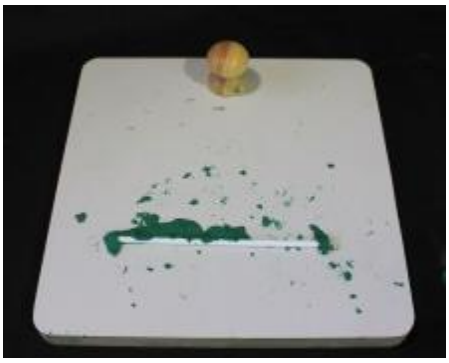<br>Level-0-construction<br>0.3 cm	
10	68	F	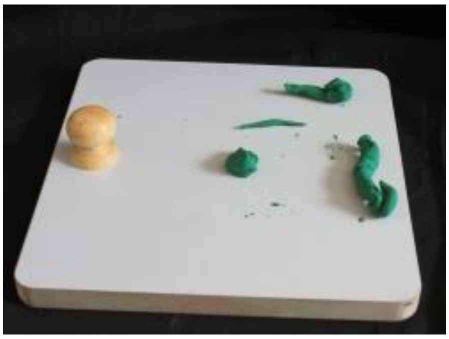<br>Plasticine only<br>2 cm	
37	50	F	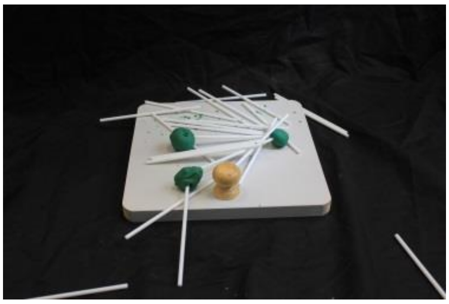<br>Level-0-construction<br>3.4 cm	

91	63	F	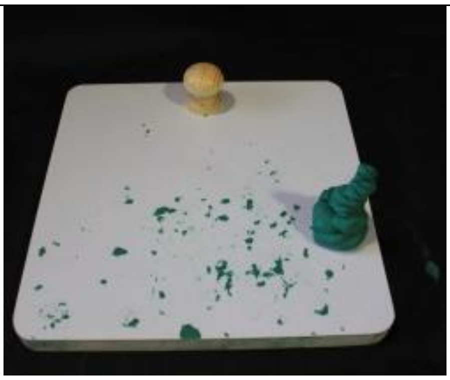<br>Plasticine tower<br>8 cm	
45	56	M	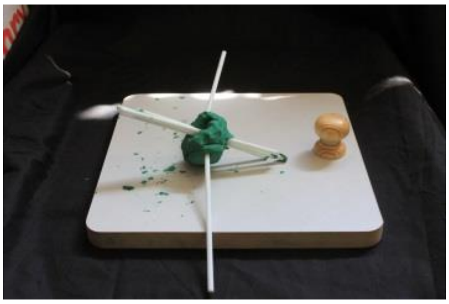<br>Level-0-construction<br>7 cm	
55	60	M	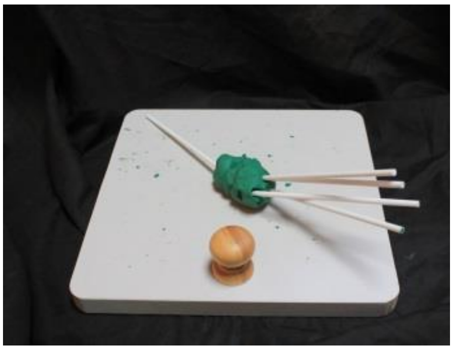<br>Level-2-tower which never stood on its own<br>10 cm	No video available<br>Star<br>17 cm
75	63	F	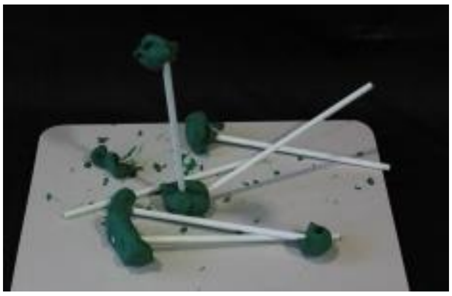<br>Level-1-tower<br>15 cm	

26	51	F	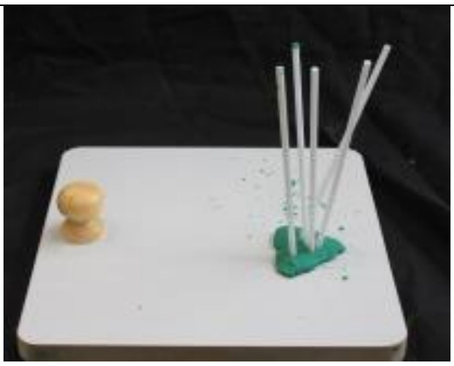<br>Level-1-tower<br>15.2 cm
52	66	M	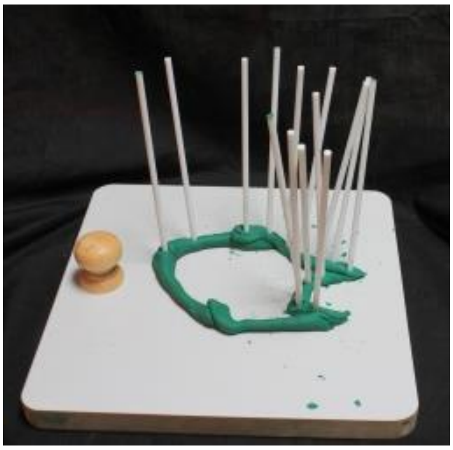<br>Level-1-tower<br>15.5 cm
32	54	F	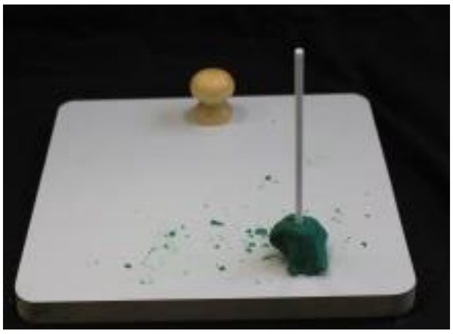<br>Level-1-tower<br>15.5 cm
46	54	F	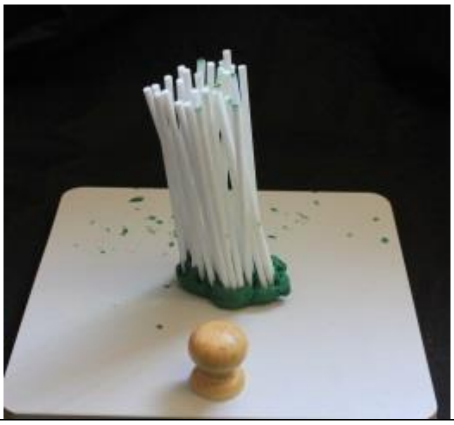

			Hedgehog<br>16 cm	
42	55	M	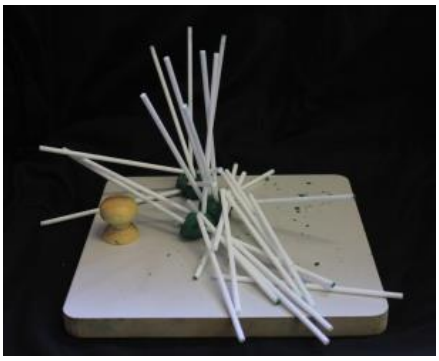<br>Hedgehog<br>16 cm	
18	64	M	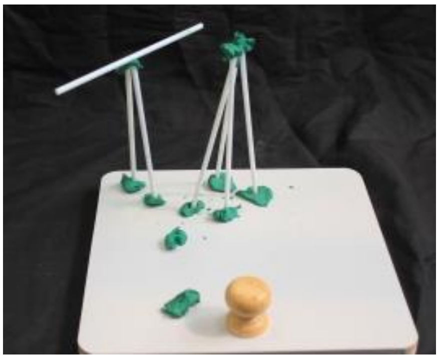<br>Level-1-tower<br>16.2 cm	
60	64	M	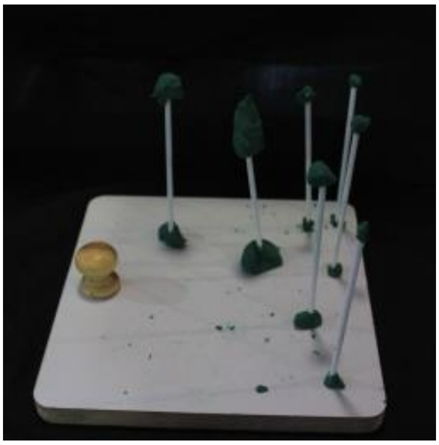<br>Level-1-tower<br>16.5 cm	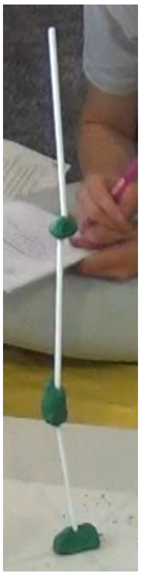<br>Level-3-tower<br>41 cm

49	63	M	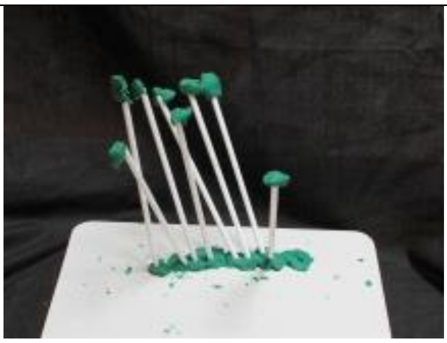 Level-1-tower 17 cm	No video available Level-2-tower 28 cm
40	58	F	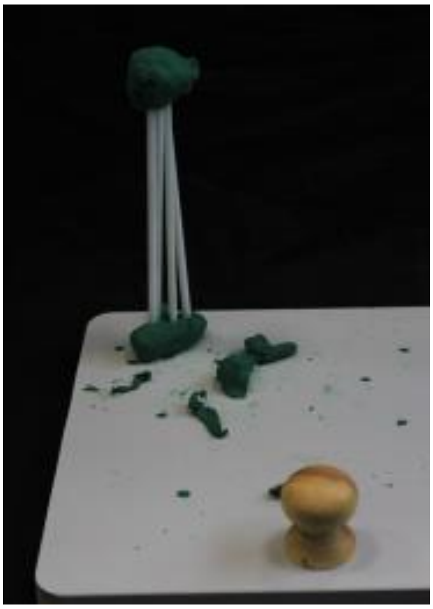 Level-1-tower 17 cm	
6	62	F	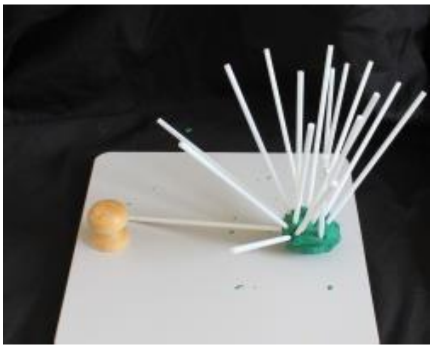 Hedgehog 17.8 cm	

92	60	F	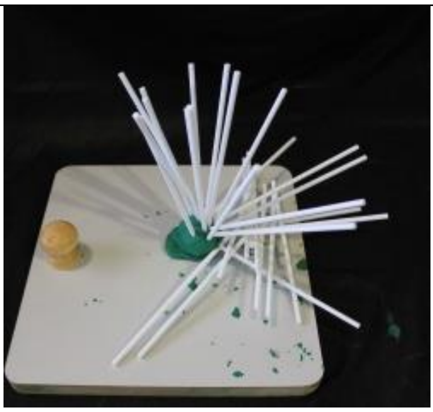 Hedgehog<br>18 cm
16	65	M	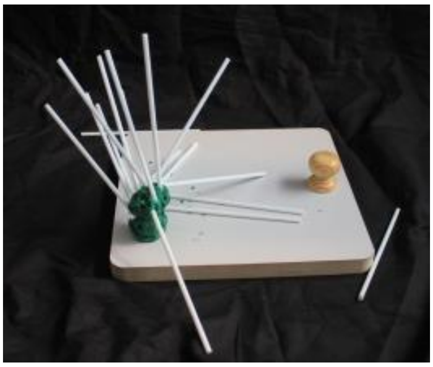 Hedgehog<br>19.7 cm
94	67	F	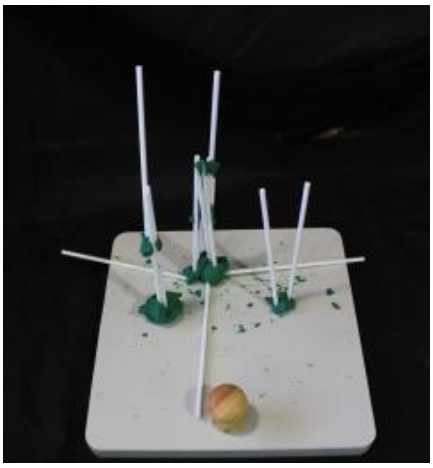 Level-2-tower<br>23 cm

23	61	M	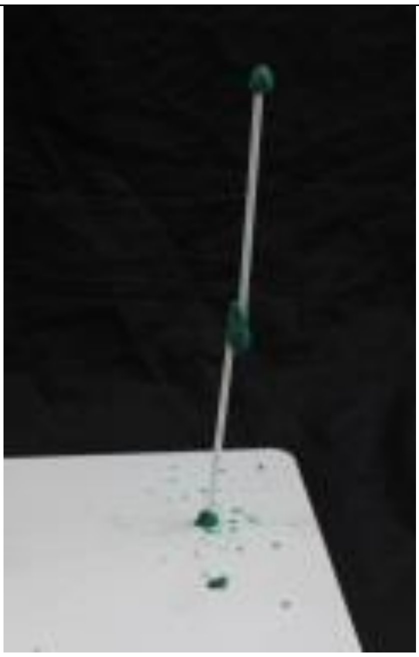<br>Level-2-tower<br>28 cm
12	57	F	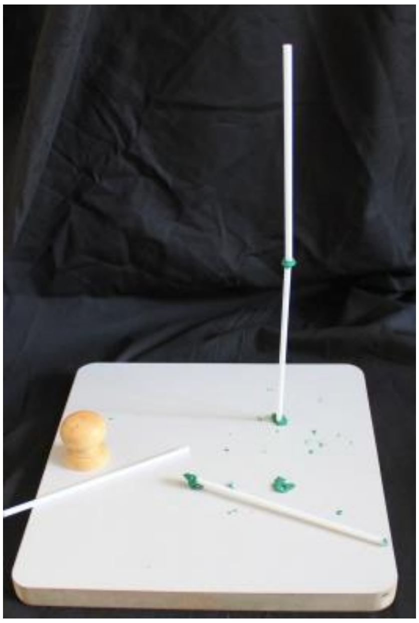<br>Level-2-tower<br>29.8 cm

20	58	M	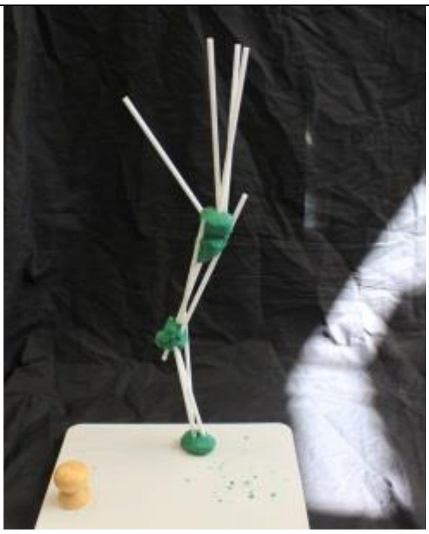<br>Level-3-tower<br>41 cm	

Table S4. *Towers of children in the full demonstration condition, sorted by height of final tower.*

**Subject**	**Age** (in months)	**Sex**	**Final tower**<br>*Picture*<br>*Shape*<br>*Height*	**Tower with maximum height** (if final tower was not the one with maximum height)<br>*Picture*<br>*Shape*<br>*Height*
47	60	M	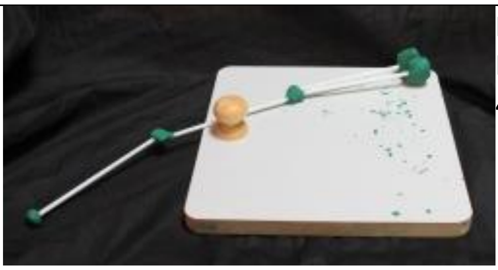<br>Crashed level-2-tower<br>3 cm	No video available<br>Level-2-tower<br>45 cm
56	63	M	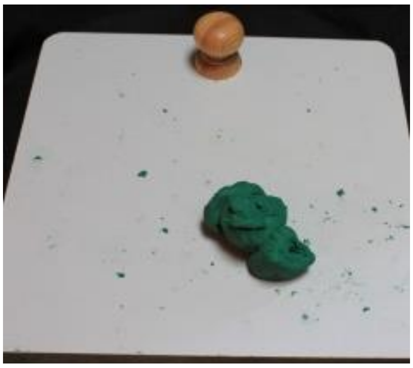<br>Plasticine only<br>3.5 cm	No video available<br>Level-2-tower<br>29 cm
86	65	M	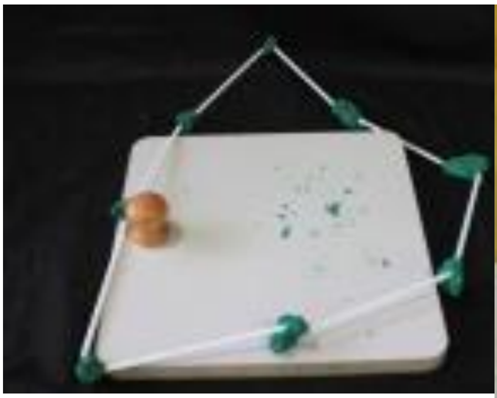<br>Level-0-construction<br>4 cm	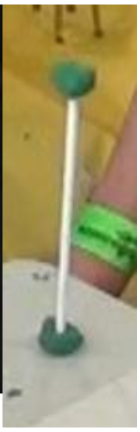<br>Level-1-tower<br>16 cm

66	60	M	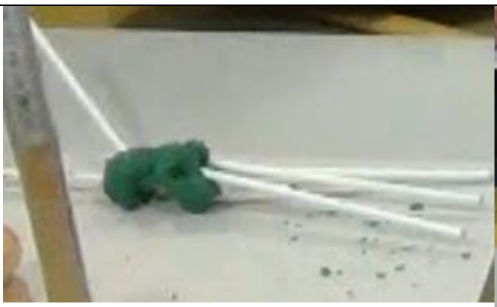<br>Star-shaped construction which never stood on its own<br>4.5 cm	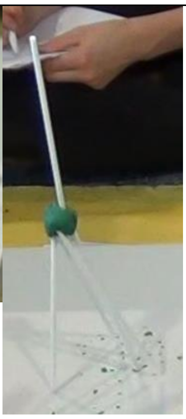<br>Star<br>27 cm
57	61	F	No picture available as child cleared table after measuring<br>Level-1-tripod<br>13 cm	No video available<br>Tripod<br>41 cm
65	63	M	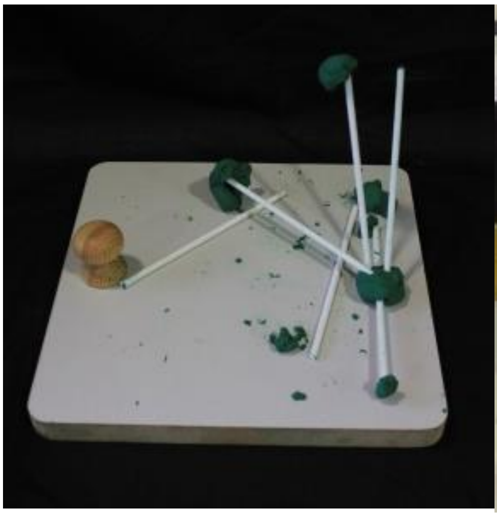<br>Level-1-tower<br>13.5 cm	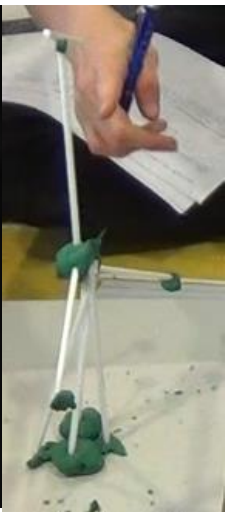<br>Modified level-2-tripod<br>32 cm
80	56	F	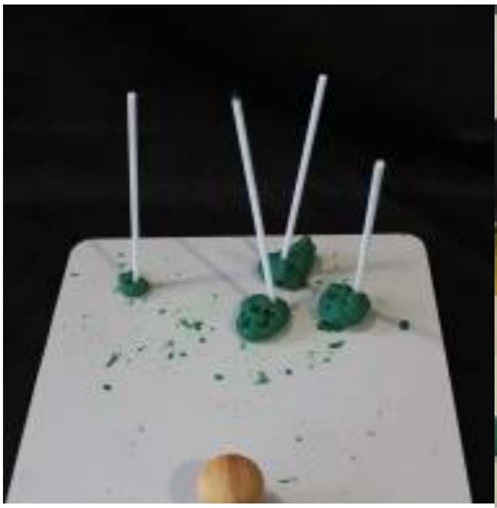<br>Level-1-tower	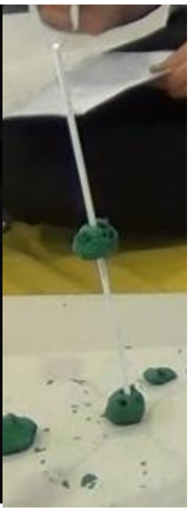<br>Level-2-tower

			15 cm	28.5 cm
43	57	M	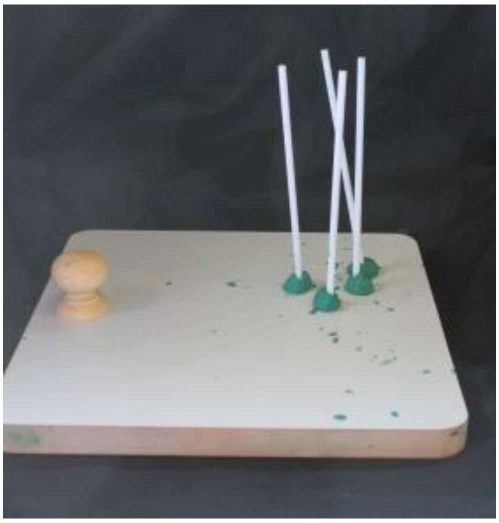 Level-1-tower<br>15 cm	
31	51	F	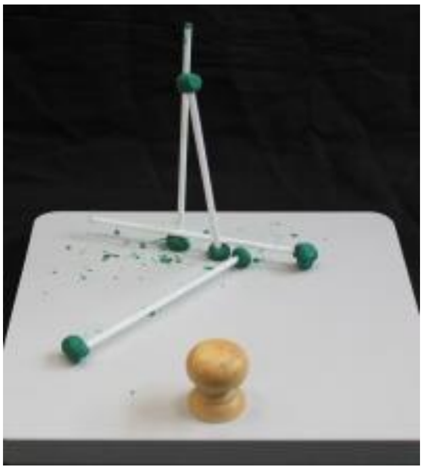 Level-1-tower<br>15 cm	
13	63	M	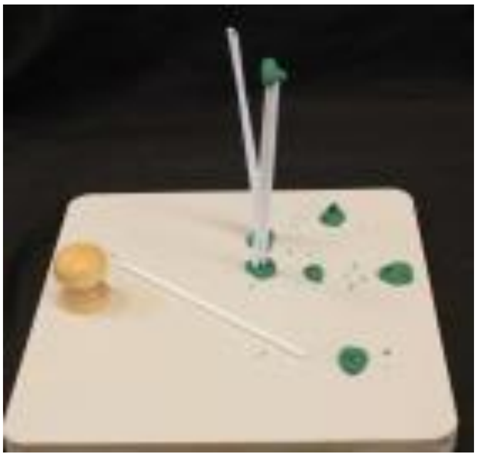 Level-1-tower<br>15.5 cm	

53	59	M	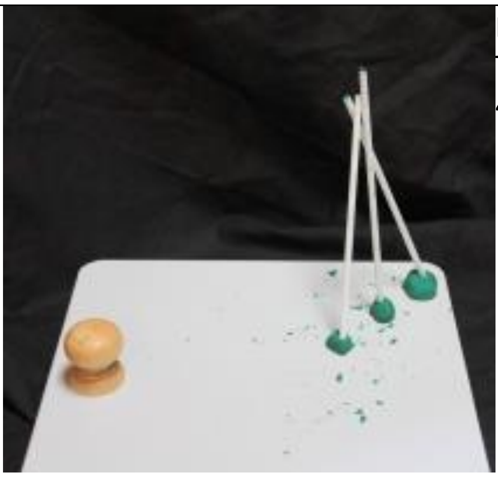<br>Level-1-tower<br>15.5 cm	No video available<br>Tripod<br>43 cm
28	57	F	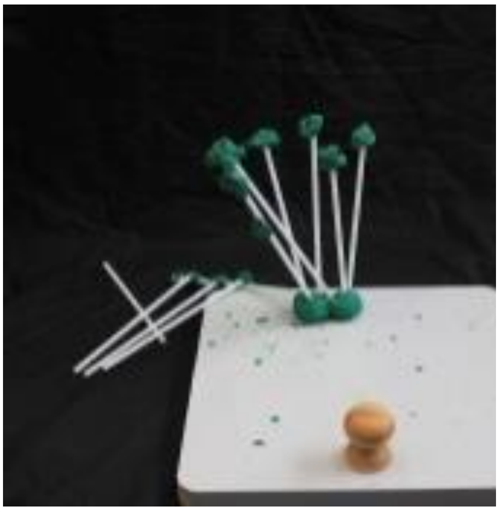<br>Hedgehog<br>15.8 cm	
38	57	F	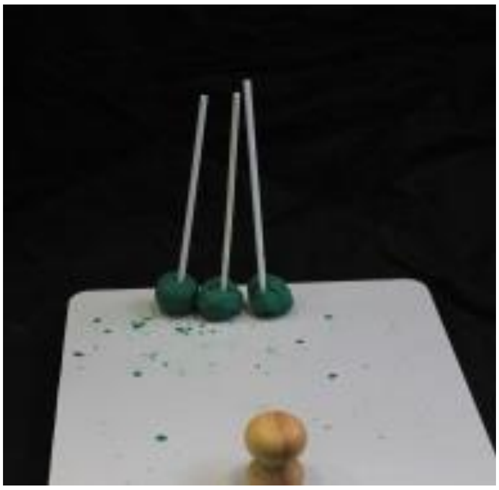<br>Level-1-tower<br>15.8 cm	

87	58	M	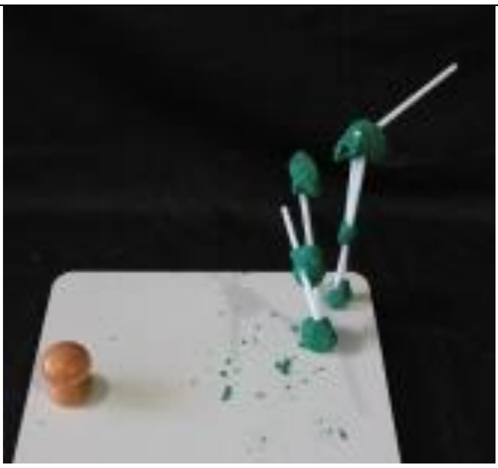<br>Level-2-tower<br>16 cm
17	60	F	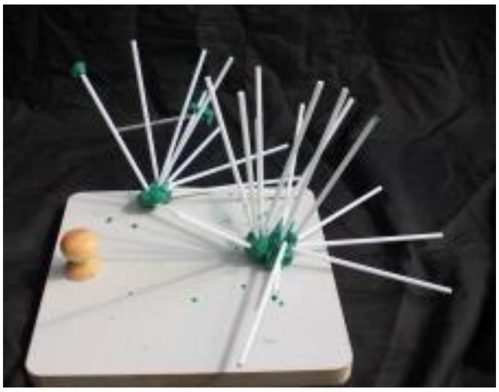<br>Hedgehog<br>16.6 cm
71	56	M	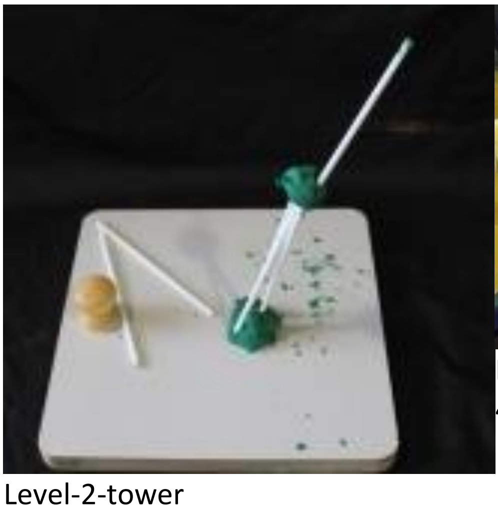<br>Level-2-tower<br>25 cm<br>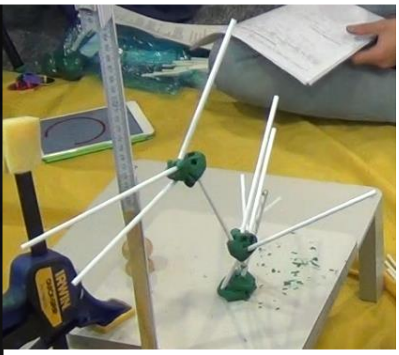<br>Level-3-tower<br>42.5 cm

51	58	M	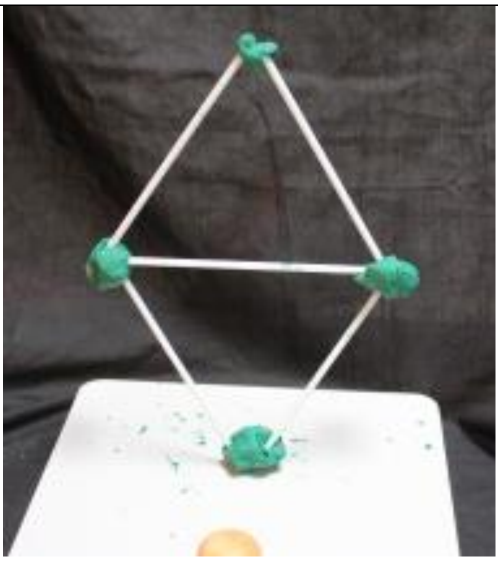<br>Diamond<br>26.5 cm
41	59	F	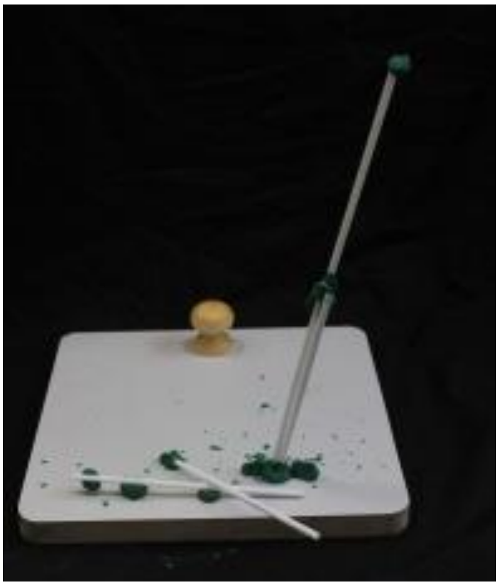<br>Level-2-tower<br>28.5 cm

62	60	M	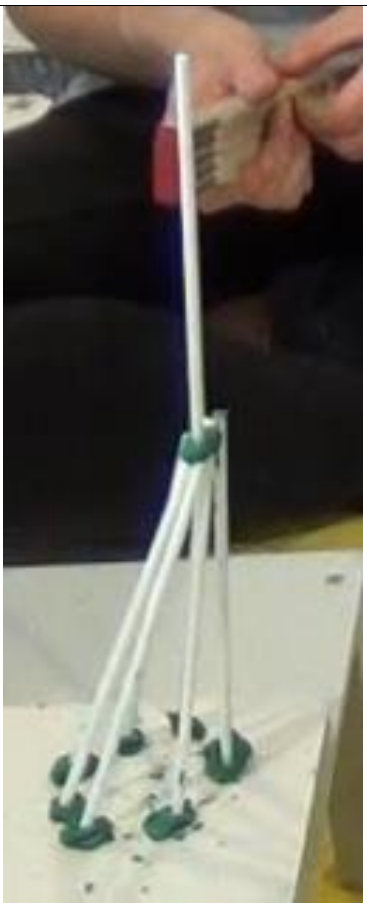 Modified level-2-tripod 29 cm
24	60	F	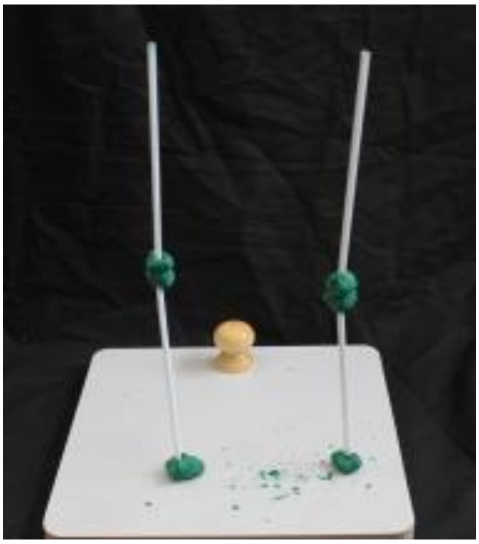 Level-2-tower 30 cm

7	61	M	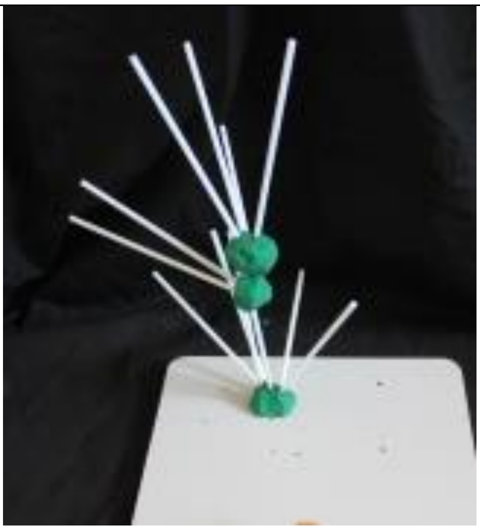<br>Level-2-tower<br>30 cm
21	63	F	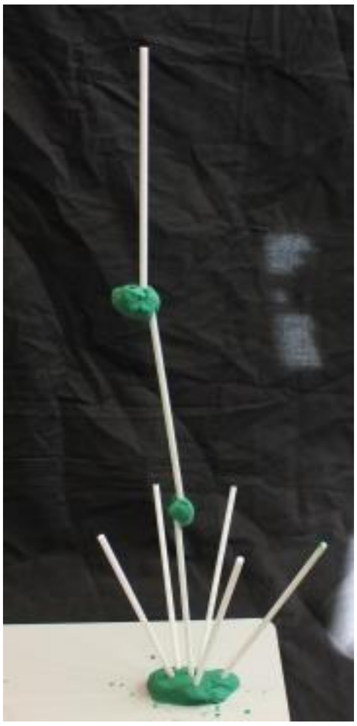<br>Level-3-tower<br>44 cm

64	58	F	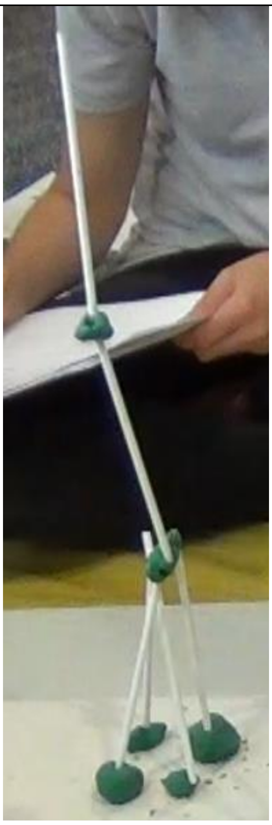<br>Tripod<br>44 cm

Table S5. *Towers of children in the endstate-only demonstration condition, sorted by height of final tower.*

Subject	Age (in months)	Sex	**Final tower**<br>*Picture*<br>*Shape*<br>*Height*	**Tower with maximum height** (if final tower was not the one with maximum height)<br>*Picture*<br>*Shape*<br>*Height*
44	53	M	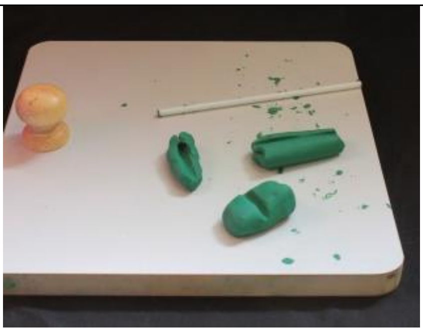<br>Plasticine tower<br>2 cm	
54	65	M	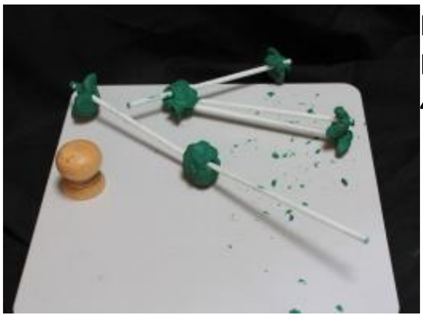<br>Tripod which never stood on its own<br>4 cm	No video available<br>Level-3-tower<br>41 cm
58	64	F	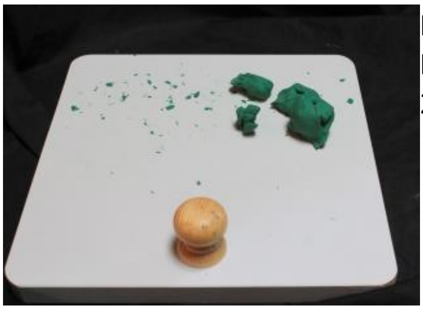<br>Crashed tripod<br>4 cm	No video available<br>Level-2-tower<br>29 cm

84	55	F	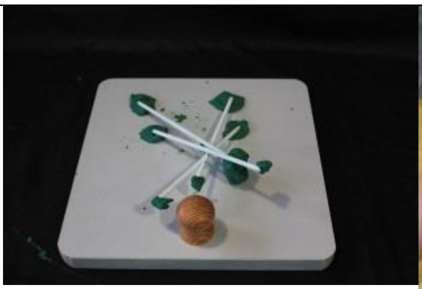<br>Crashed level-1-tower<br>6 cm	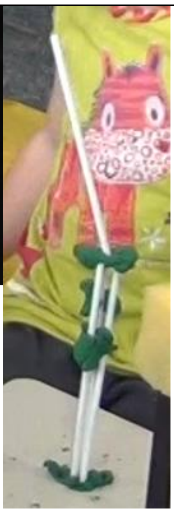<br>Level-2-tower<br>29 cm
48	64	M	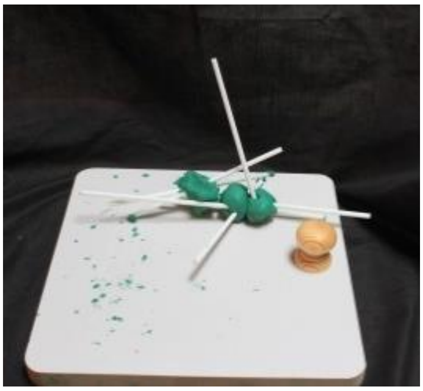<br>Level-1-tower<br>14 cm	
59	57	M	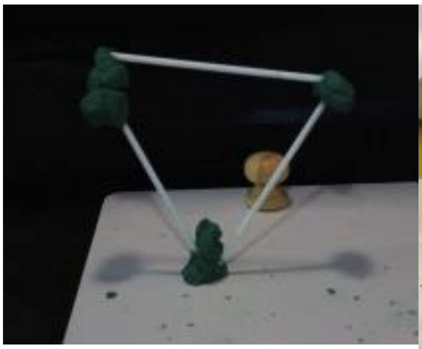<br>Level-1-triangle<br>14.5 cm	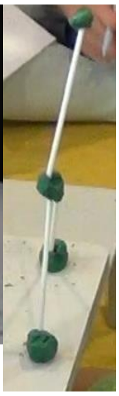<br>Level-2-tower<br>28 cm

73	53	F	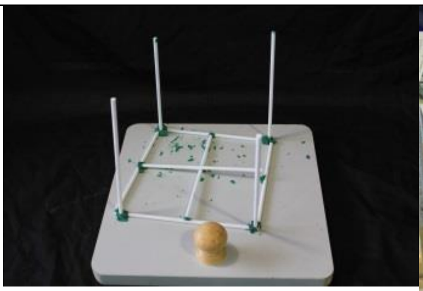 Level-1-cube<br>15 cm	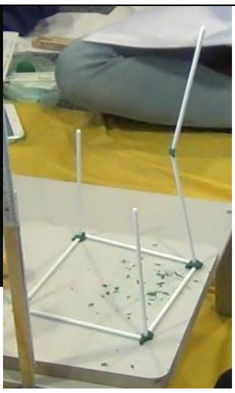 Level-2-cube<br>29 cm
1	61	F	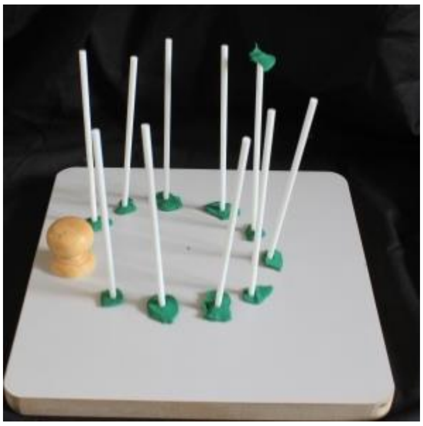 Level-1-tower<br>15 cm	
67	57	M	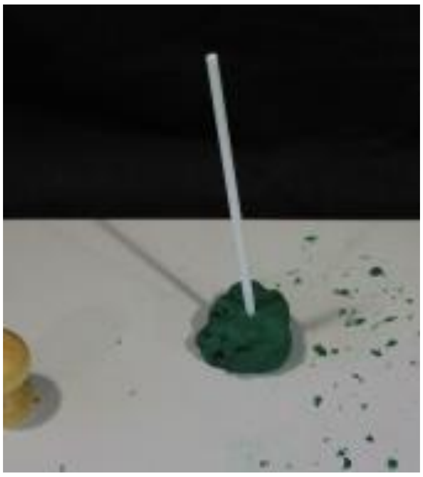 Level-1-tower<br>15 cm	

90	54	F	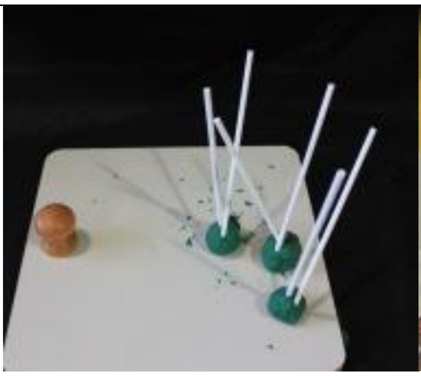<br>Level-1-tower<br>15.5 cm	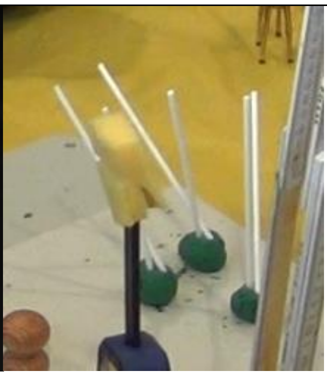<br>Level-1-tower<br>17 cm
63	56	M	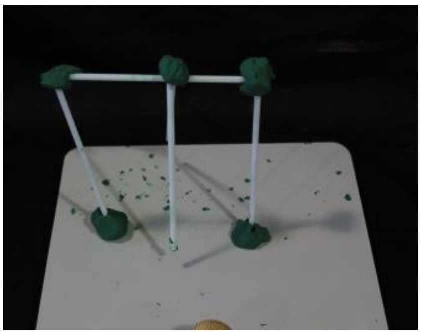<br>Level-1-square<br>17 cm	
27	58	M	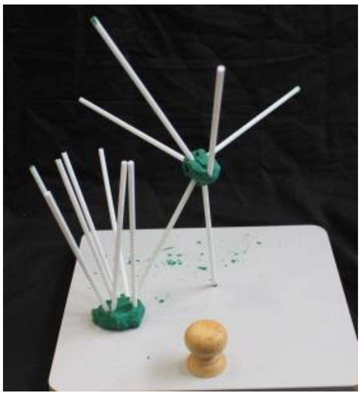<br>Star<br>21 cm	
69	63	M	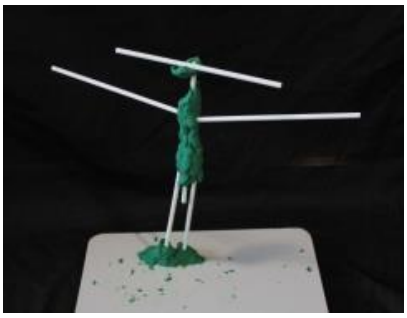<br>Level-2-tower<br>23 cm	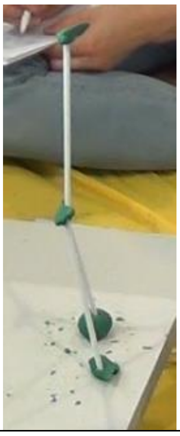

				Level-2- tower<br>29 cm
72	64	F	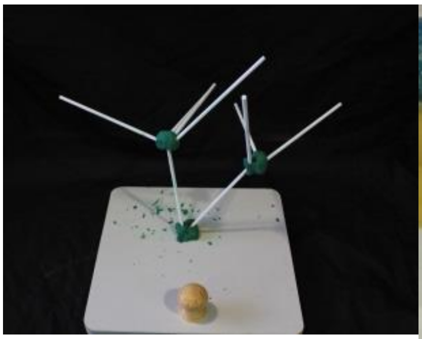<br>Level-2-tower<br>24.5 cm	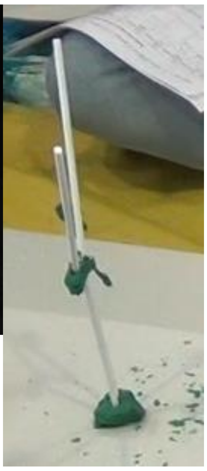<br>Level-2-tower<br>26 cm
89	63	F	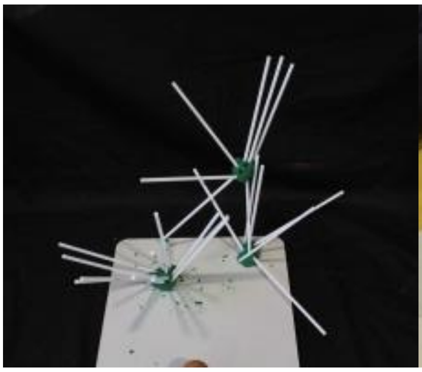<br>Level-2-tower<br>24.7 cm	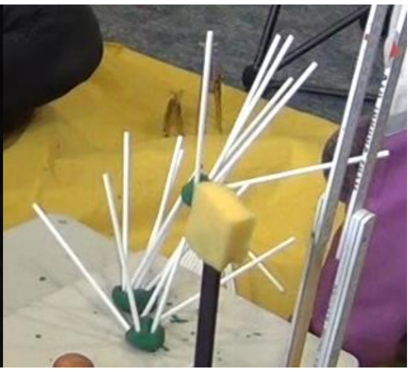<br>Level-2-tower<br>27.5 cm
36	51	F	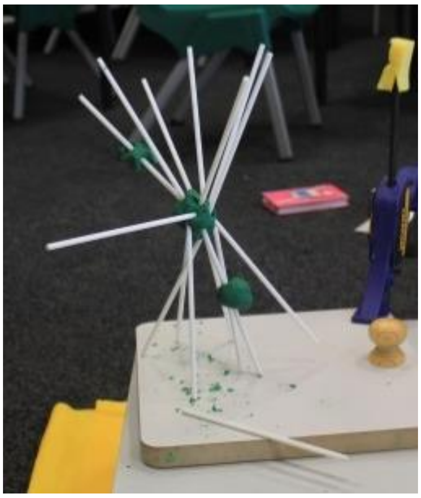<br>Star<br>28 cm	

74	68	F	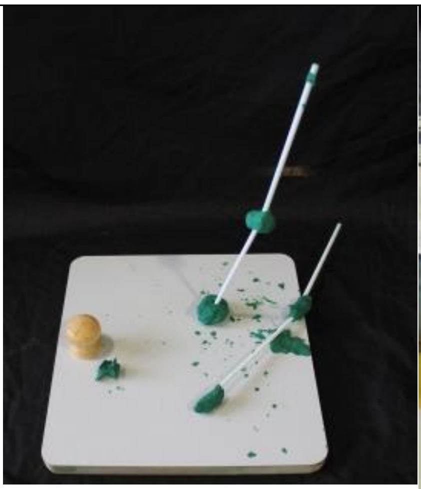<br>Level-2-tower<br>28 cm	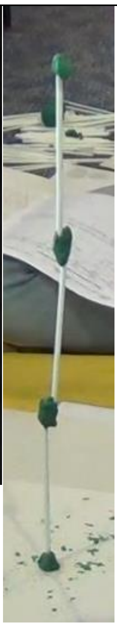<br>Level-3-tower<br>42 cm
14	63	M	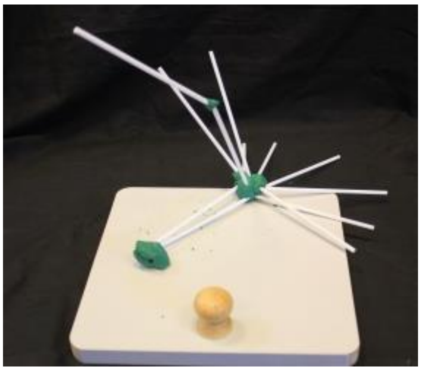<br>Level-2-tower<br>28.5 cm	
88	60	F	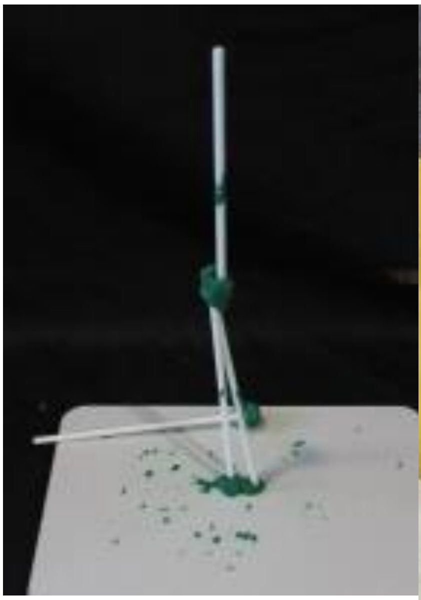<br>Level-2-tower<br>29 cm	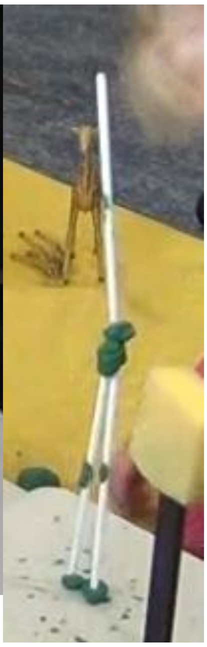<br>Level-2-tower

				30 cm
81	67	F	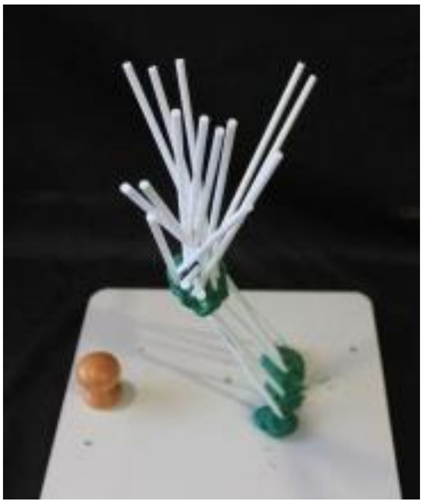<br>Level-2-tripod<br>29 cm	
85	64	F	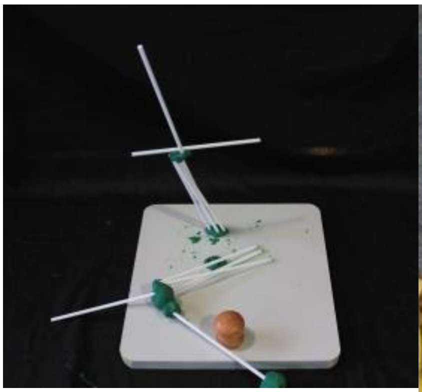<br>Level-2-tower<br>29.5 cm	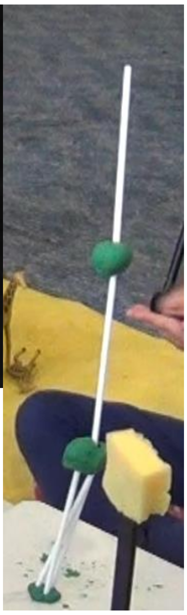<br>Level-3-tower<br>41 cm
83	65	F	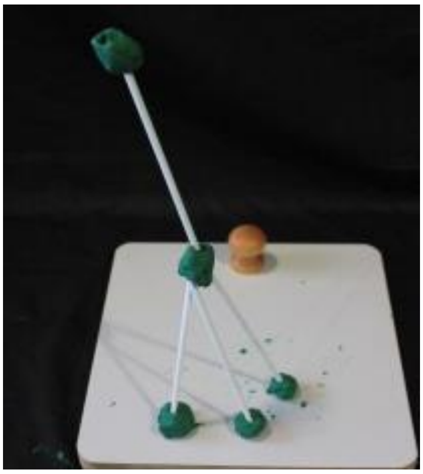<br>Level-2-tripod	

			30.5 cm
50	59	M	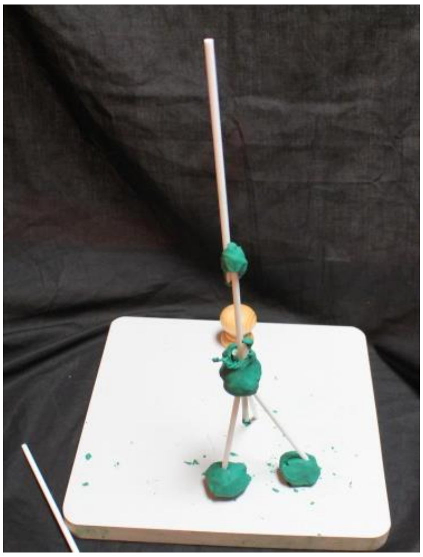<br>Level-3-tower<br>33.7 cm
82	60	M	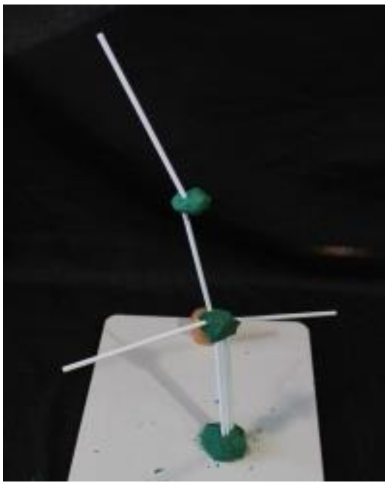<br>Level-3-tower<br>38 cm
68	63	F	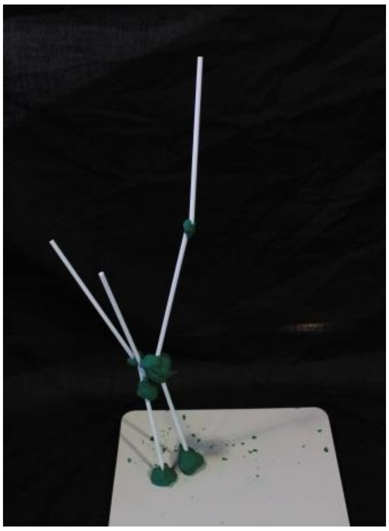<br>Level-3-tower

			43 cm
77	57	F	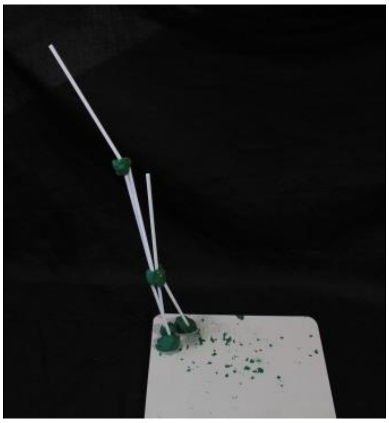 Tripod 44cm
29	65	F	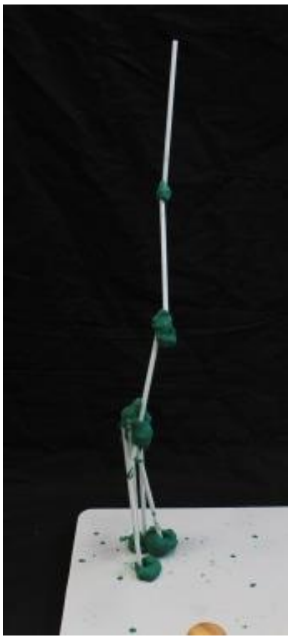 Level-4-tripod 45.5cm

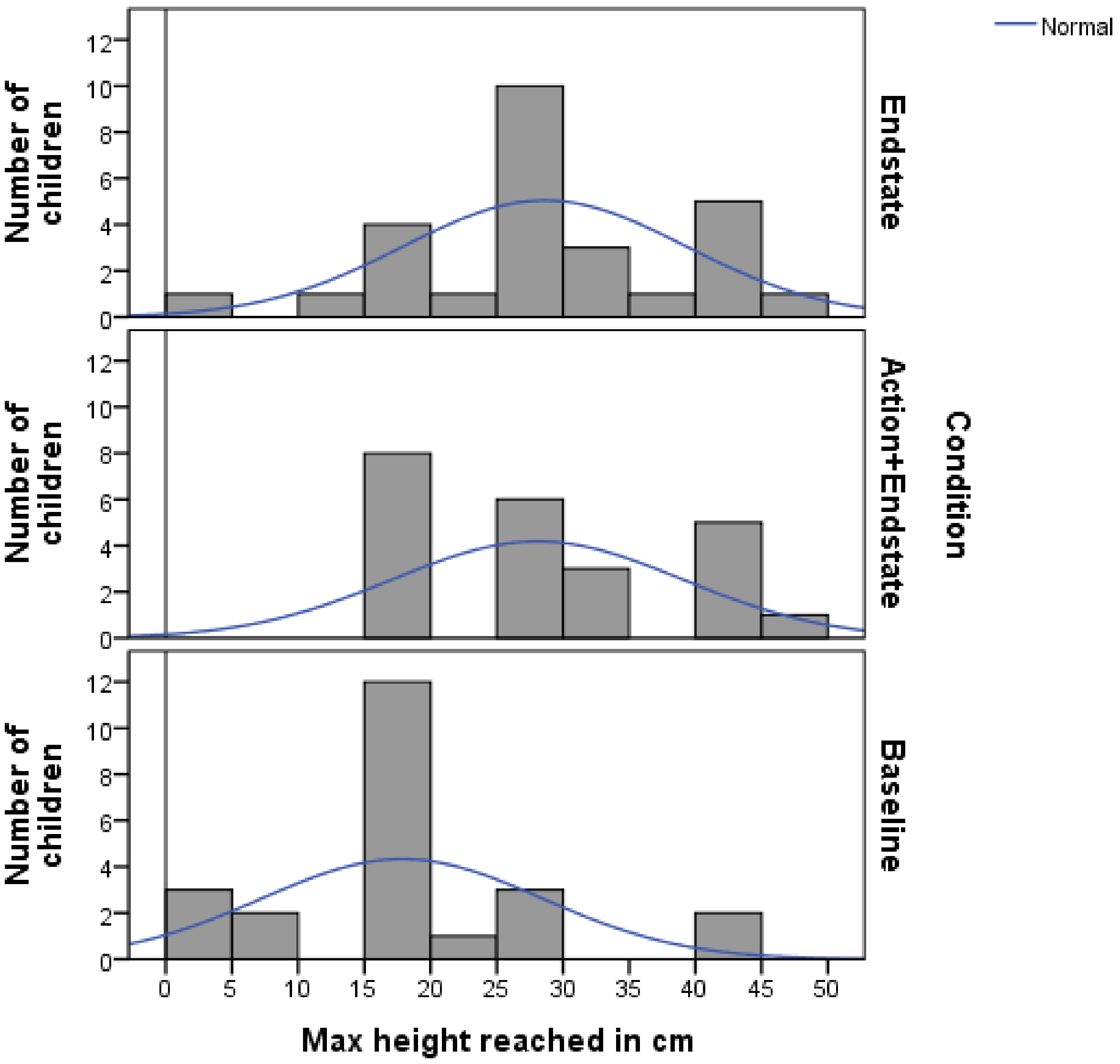

Fig. S1. Maximum tower height reached in the baseline, full demonstration (i.e., Action + Endstate), and Endstate-only demo.

Supplementary results: Main study

Our results showed that the most common shape across conditions was what we called “towers”, i.e., constructions standing on a single “leg”. Whilst most children in the baseline condition built level-1-towers (a single stick standing upright), the most common tower shape in the demo conditions was a level-2-tower (two sticks joined on top of each other). In addition, we found that these towers could reach considerable heights: for example, the two tallest towers in the baseline condition were both 41 cm tall. Does this mean that the tripod is not such a beneficial shape at all and that baseline children – who cannot draw on the tripod design – can complete the task equally well by building towers? Data from previous studies[7] and our own work indicate that this is not the case.

First, Caldwell and colleagues[7] report that the tripod is a “typical tower type[s]”[(p164)] found in their spaghetti tower studies conducted with adults who chose their designs freely and spontaneously. Our own informal observations of older children (7 years and above) as well as adults doing the task at our outreach events indicate that older/more experienced individuals prefer to build constructions with three or even four legs (the Eiffeltower shape) and thereby are able to reach heights up to around 70 cm. These observations indicate that the tripod design is efficient for the task.

Second, our own results show that even though the towers in the baseline condition reached a height up to 41 cm, this was a rare incidence: even though half of the baseline children in the main study (and 3 out of 17 in Pilot study 2) built towers, only 2 out of 23 baseline children in the main study were able to make towers taller than 30 cm (and potentially 1 out of 17 in the baseline of Pilot study 2 (but there is no exact measurement as this tower was disassembled before the end of the trial)). We visualized these data for the case of the main study in Fig. S2 which shows that only a small part of the towers were able to

reach heights greater than 30 cm. In contrast, the tripods were all greater than 30 cm (and actually even taller than 40 cm). This means that the strategy of building towers was not very efficient, i.e., the "success rate" of building towers was rather low (see Table S5 and a visualization in Fig. S3): Out of all towers built, only between 16.67% (baseline) and 38.89% (endstate-only condition) reached heights taller than 30 cm. The success rates of (modified) tripods, however, were at 75% (endstate-only condition) and 80% (full demo condition).

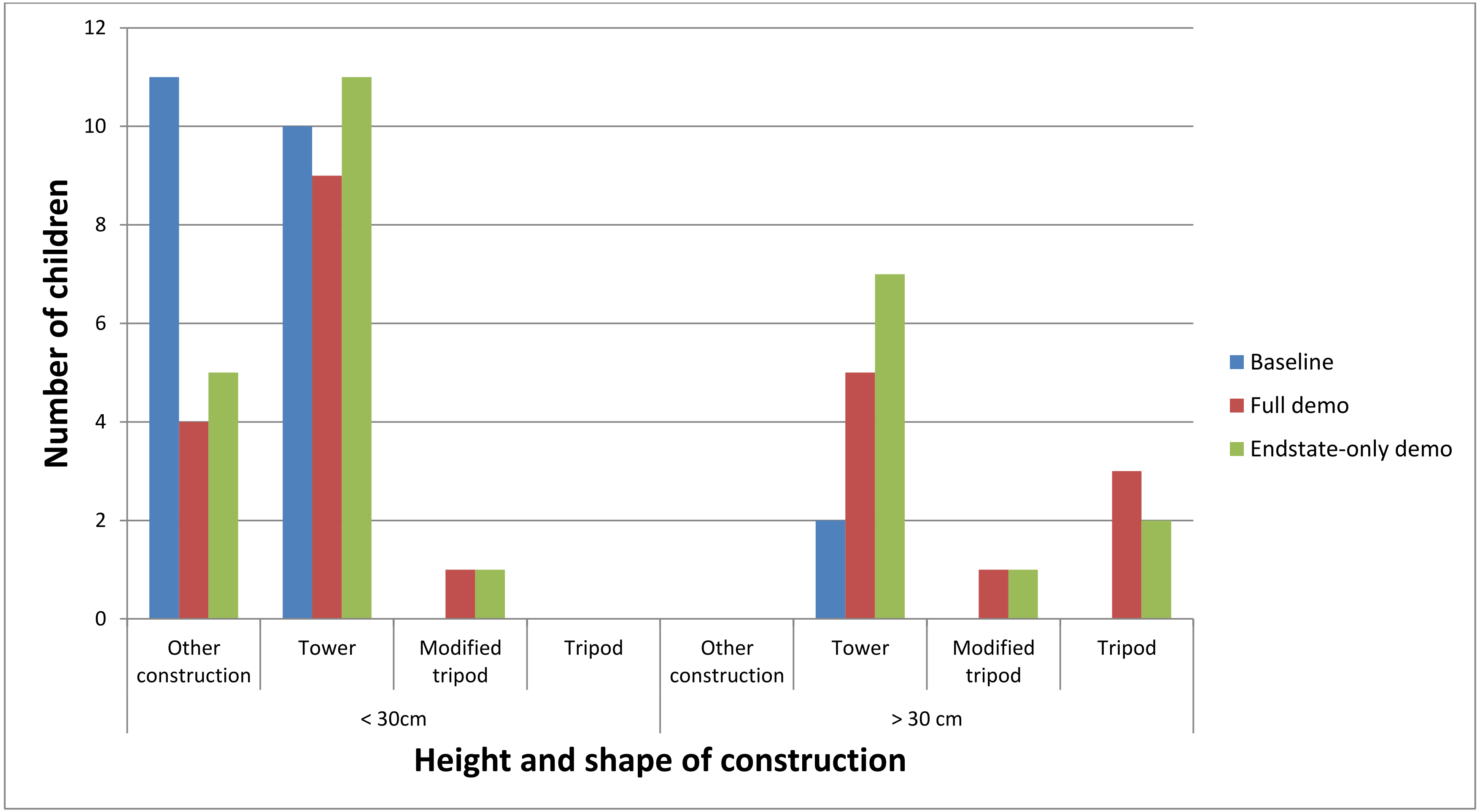

Fig. S2. Height and shape of the constructions in the three conditions of the main study.

Table S6. *"Success rates" of towers and (modified) tripods in the three conditions of the main study.*

	Condition		
	Endstate-only	Full demo	Baseline
Total number of constructions	27	23	23
Thereof towers	18 (66.67%)	14 (60.87%)	12 (52.17%)
Out of the towers: those > 30cm (success rate)	7 (38.89%)	5 (35.71%)	2 (16.67%)
Thereof tripods or modified tripods	4 (14.81%)	5 (21.74%)	0
Out of the (modified) tripods: those > 30cm (success rate)	3 (75.0%)	4 (80.0%)	0

Note. Success rate is defined as the proportion of towers taller than 30cm out of all towers built or, respectively, the proportion of (modified) tripods taller than 30 cm out of all (modified) tripods built for each condition.

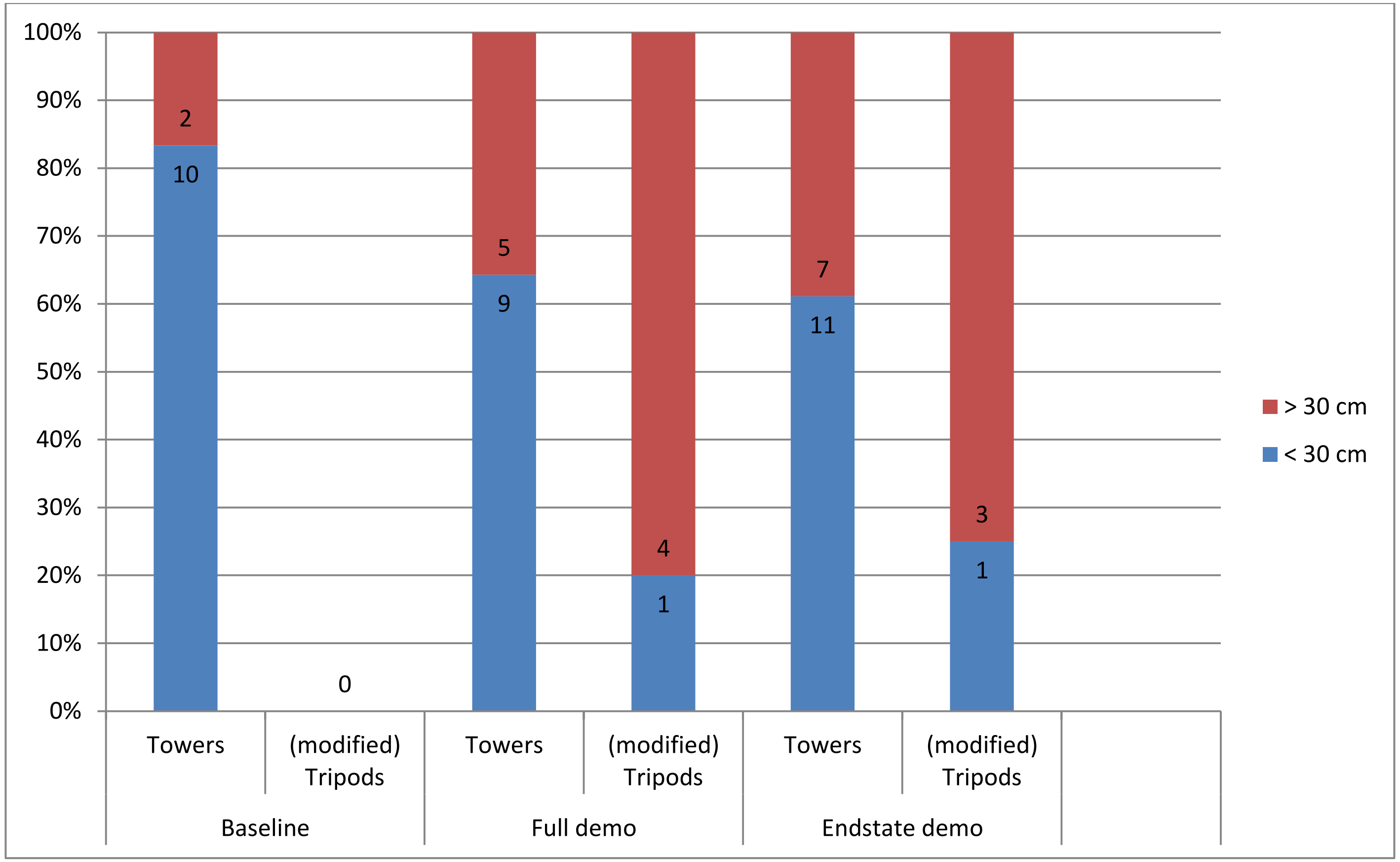

Fig. S3. Proportion of towers and (modified) tripods greater/smaller than 30 cm, separated by condition.
